# Supplementary material for: Suppression of upstream ORF translation is not a widespread mechanism of translational stimulation by yeast helicase Ded1
Source: bioRxiv. 2026 Apr 11:2026.04.10.717766. Preprint. [Version 1] doi: 10.64898/2026.04.10.717766 (PMC13082114; doi:10.64898/2026.04.10.717766)
Supplement: Supplement 1 [file NIHPP2026.04.10.717766v1-supplement-1.pdf]

bioRxiv preprint doi: https://doi.org/10.64898/2026.04.10.717766; this version posted April 11, 2026. The copyright holder for this preprint (which was not certified by peer review) is the author/funder. This article is a US Government work. It is not subject to copyright under 17 USC 105 and is also made available for use under a CC0 license.

SUPPLEMENTARY MATERIAL FOR:

Suppression of upstream ORF translation is not a widespread mechanism of translational stimulation by yeast helicase Ded1

Rakesh Kumar[1], Gemma May[2], Neelam Dabas Sen[1,3†], C. Joel McManus[2,4†] and Alan G. Hinnebusch[1†]

bioRxiv preprint doi: https://doi.org/10.64898/2026.04.10.717766; this version posted April 11, 2026. The copyright holder for this preprint (which was not certified by peer review) is the author/funder. This article is a US Government work. It is not subject to copyright under 17 USC 105 and is also made available for use under a CC0 license.

SUPPLEMENTARY FIGURES

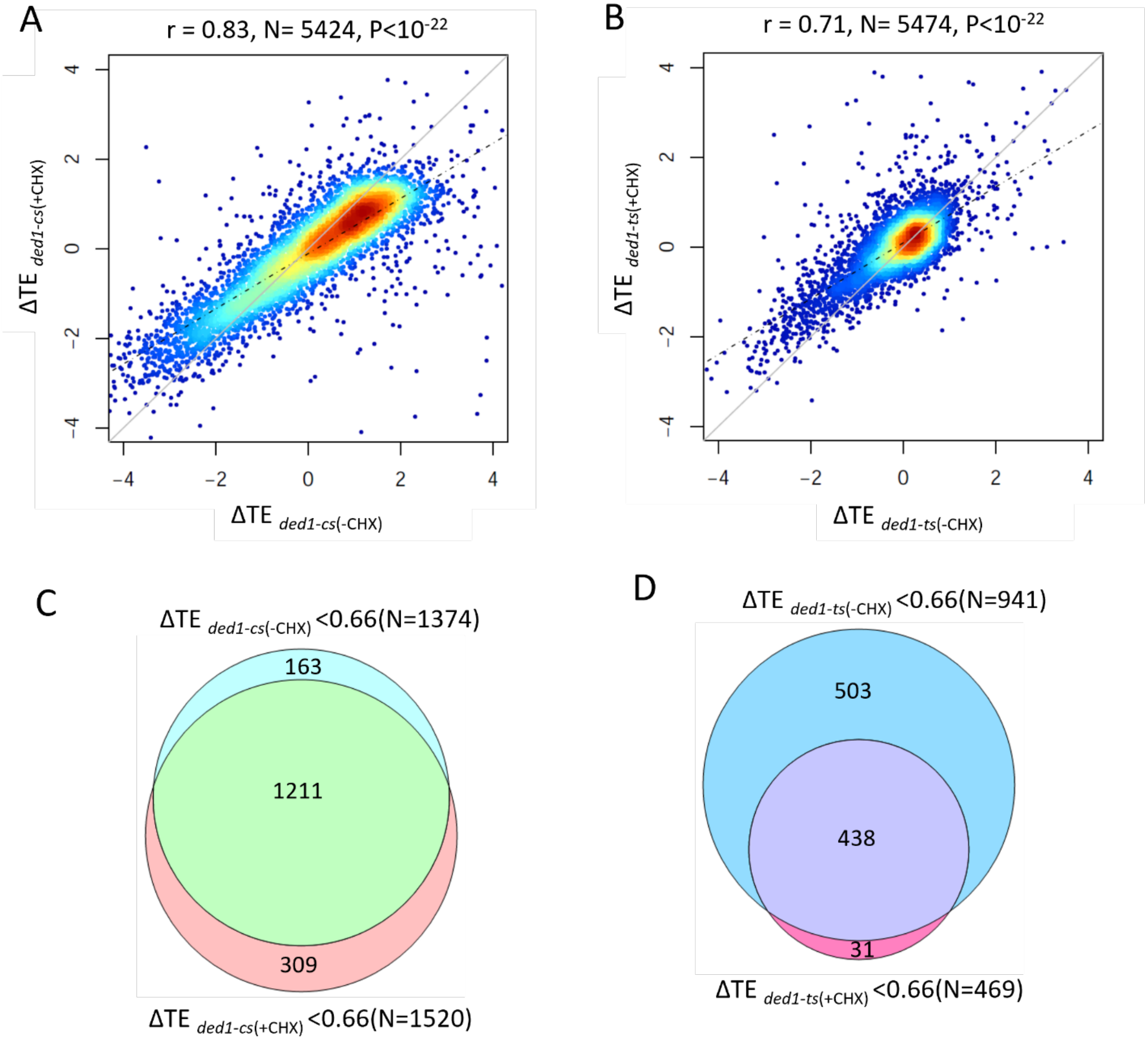

Figure S1. Genome-wide translational changes of mORFs conferred by *ded1-cs* and *ded1-ts* mutations are influenced minimally by treatment of cells with cycloheximide

(A) Density plot of $\log_2$ fold-changes in TE for mORFs conferred by *ded1-cs* versus *DED1* for cells treated (+CHX) or untreated with CHX (-CHX), for 5,424 expressed genes, excluding from the plot (but not the analysis) genes with $-6 > \log_2\Delta TE > 6$ values to expand the axes. The dotted

bioRxiv preprint doi: https://doi.org/10.64898/2026.04.10.717766; this version posted April 11, 2026. The copyright holder for this preprint (which was not certified by peer review) is the author/funder. This article is a US Government work. It is not subject to copyright under 17 USC 105 and is also made available for use under a CC0 license.

line is the determined regression line; grey solid line is the theoretical regression line for identical changes in ΔTE values. Individual genes are shown by blue filled circles; the coloring indicates increased density of genes (red is maximum). TE change values of +CHX samples were taken from (1). Pearson correlation coefficients (r) and associated P-values are indicated. **(B)** Similar to (A), but for 5,474 expressed genes quantified in the *ded1-ts*(+CHX) versus *ded1-ts*(-CHX) experiments. **(C)** Overlap between mRNAs exhibiting ≥1.5-fold decreases in TE at FDR<0.05 in *ded1-cs* versus *DED1* cells under CHX -treated or -untreated conditions. **(D)** Similar to (C), but for mRNAs exhibiting significant TE reductions in response to the *ded1-ts* mutation.

bioRxiv preprint doi: https://doi.org/10.64898/2026.04.10.717766; this version posted April 11, 2026. The copyright holder for this preprint (which was not certified by peer review) is the author/funder. This article is a US Government work. It is not subject to copyright under 17 USC 105 and is also made available for use under a CC0 license.

A

Avg total reads (in million)	+CHX	-CHX
WT_ts	46.2	121.6
ded1-ts	19.4	112.6
WT_cs	20.8	54.7
ded1-cs	10.3	52.3

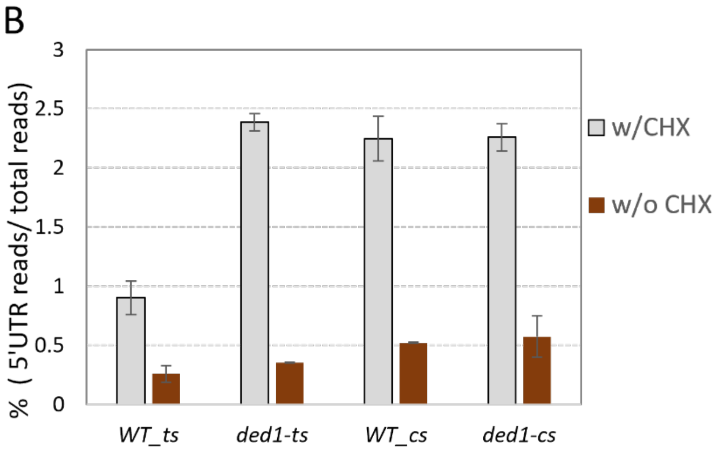

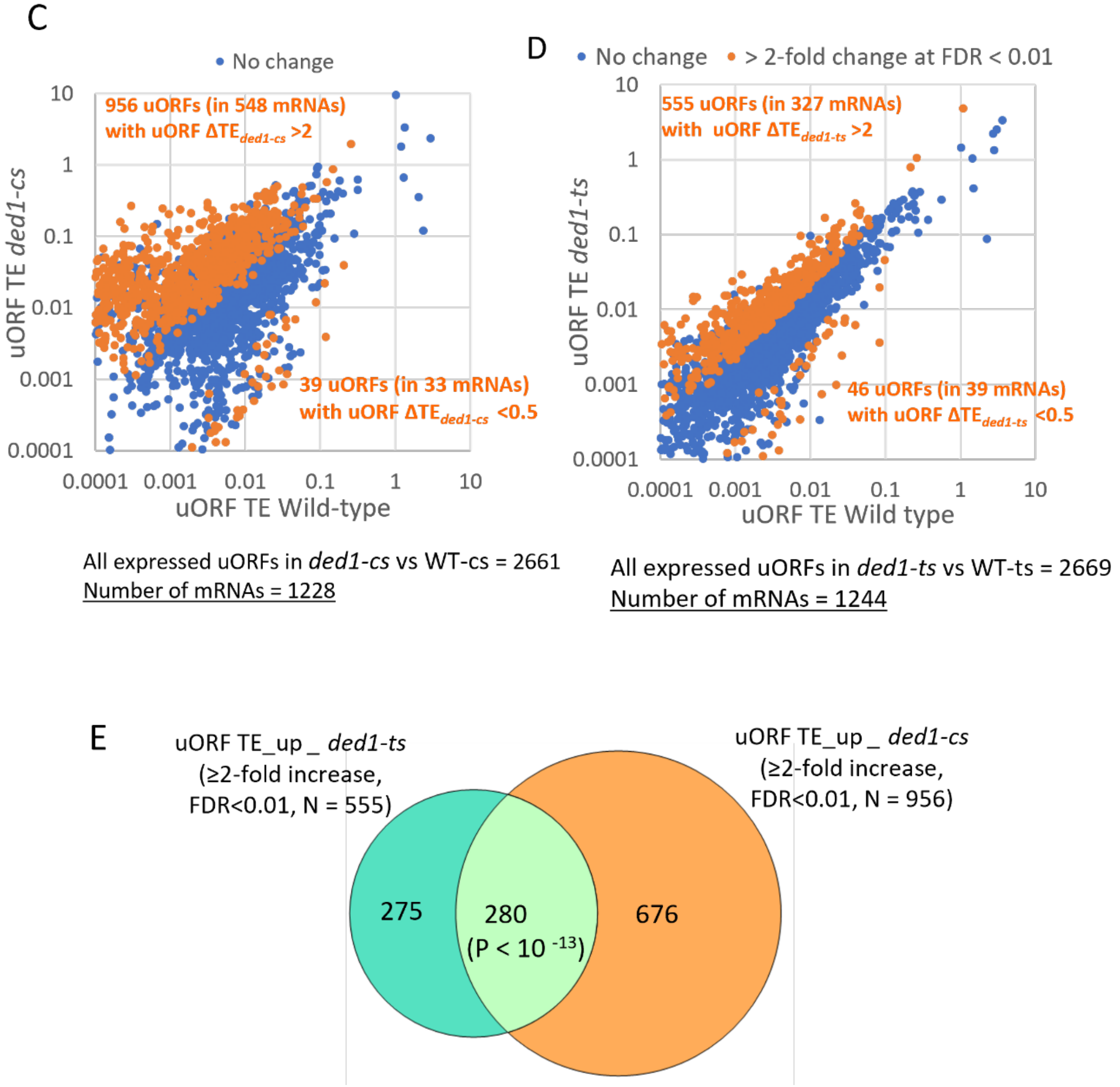

Figure S2. Despite reduced overall RPF occupancies in 5'UTRs, a significant number of mRNAs show evidence of increased uORF translation in *ded1* cells in -CHX vs. +CHX

bioRxiv preprint doi: https://doi.org/10.64898/2026.04.10.717766; this version posted April 11, 2026. The copyright holder for this preprint (which was not certified by peer review) is the author/funder. This article is a US Government work. It is not subject to copyright under 17 USC 105 and is also made available for use under a CC0 license.

experiments. **(A)** Average number of reads mapped to the yeast transcriptome from the two replicates for the indicated *ded1* mutants and the isogenic *DED1* strains from -CHX and +CHX experiments. **(B)** Fraction of total 80S RPFs mapped to 5'UTRs in the *ded1-ts, ded1-cs,* and respective *DED1* samples from cultures treated (grey) or untreated (maroon) with CHX, plotting data from two biological replicates for each strain. **(C-D)** Scatterplots of uORF TEs in the indicated mutant versus corresponding *DED1* strain from -CHX experiments for (C) *ded1-cs*, N=2661, or (C) *ded1-ts*, N=2669, with N equaling the total number of expressed uORFs analyzed in each comparison. uORF TE values were determined by DESeq2 using biological replicates of each strain for genes having ≥ 128 mRNA and ≥ 8 RPF reads in uORFs in the four samples (two replicates each of *DED1* and *ded1* mutant). Genes exhibiting ≥2-fold changes in uORF TE in *ded1* mutant versus *DED1* cells at FDR < 0.01 are highlighted in orange and the number of uORFs and mRNAs exhibiting a substantial increase or decrease in uORF TE are indicated in orange type. **(E)** Venn diagram of overlap between uORFs exhibiting ≥2-fold increases in TE in *ded1-cs* or *ded1-ts* vs. *DED1* cells., with the P value for significance of overlap based on the hypergeometric distribution.

bioRxiv preprint doi: https://doi.org/10.64898/2026.04.10.717766; this version posted April 11, 2026. The copyright holder for this preprint (which was not certified by peer review) is the author/funder. This article is a US Government work. It is not subject to copyright under 17 USC 105 and is also made available for use under a CC0 license.

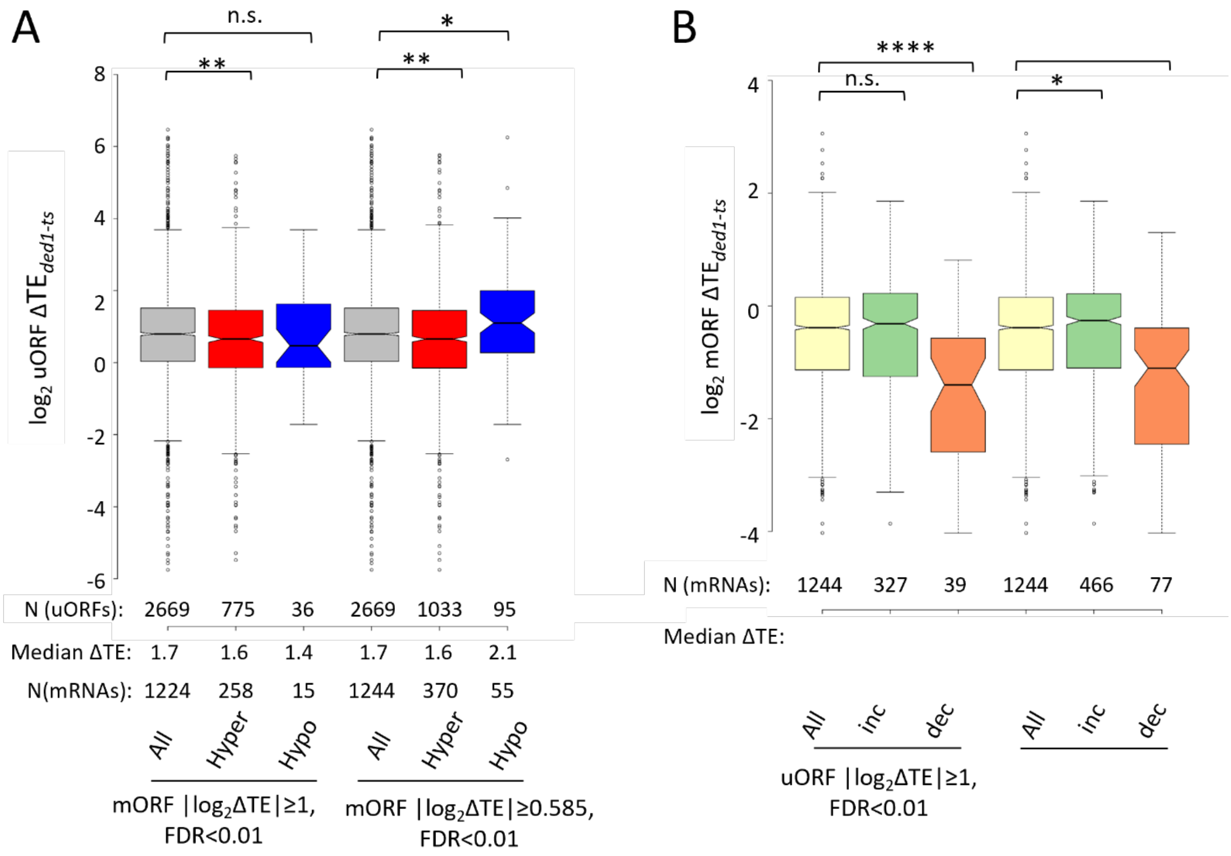

Figure S3. Increased uORF translation in *ded1-ts* cells is generally not accompanied by reduced translation of the downstream mORFs. (A)-(B) Analysis identical to Figs. 2A & C but conducted using *ded1-ts* (-CHX) Ribo-Seq data. Results of Mann-Whitney U-tests are summarized as: ****, P<0.0001; **, P<0.01; *, P<0.05; n.s., not significant, P>0.05.

bioRxiv preprint doi: https://doi.org/10.64898/2026.04.10.717766; this version posted April 11, 2026. The copyright holder for this preprint (which was not certified by peer review) is the author/funder. This article is a US Government work. It is not subject to copyright under 17 USC 105 and is also made available for use under a CC0 license.

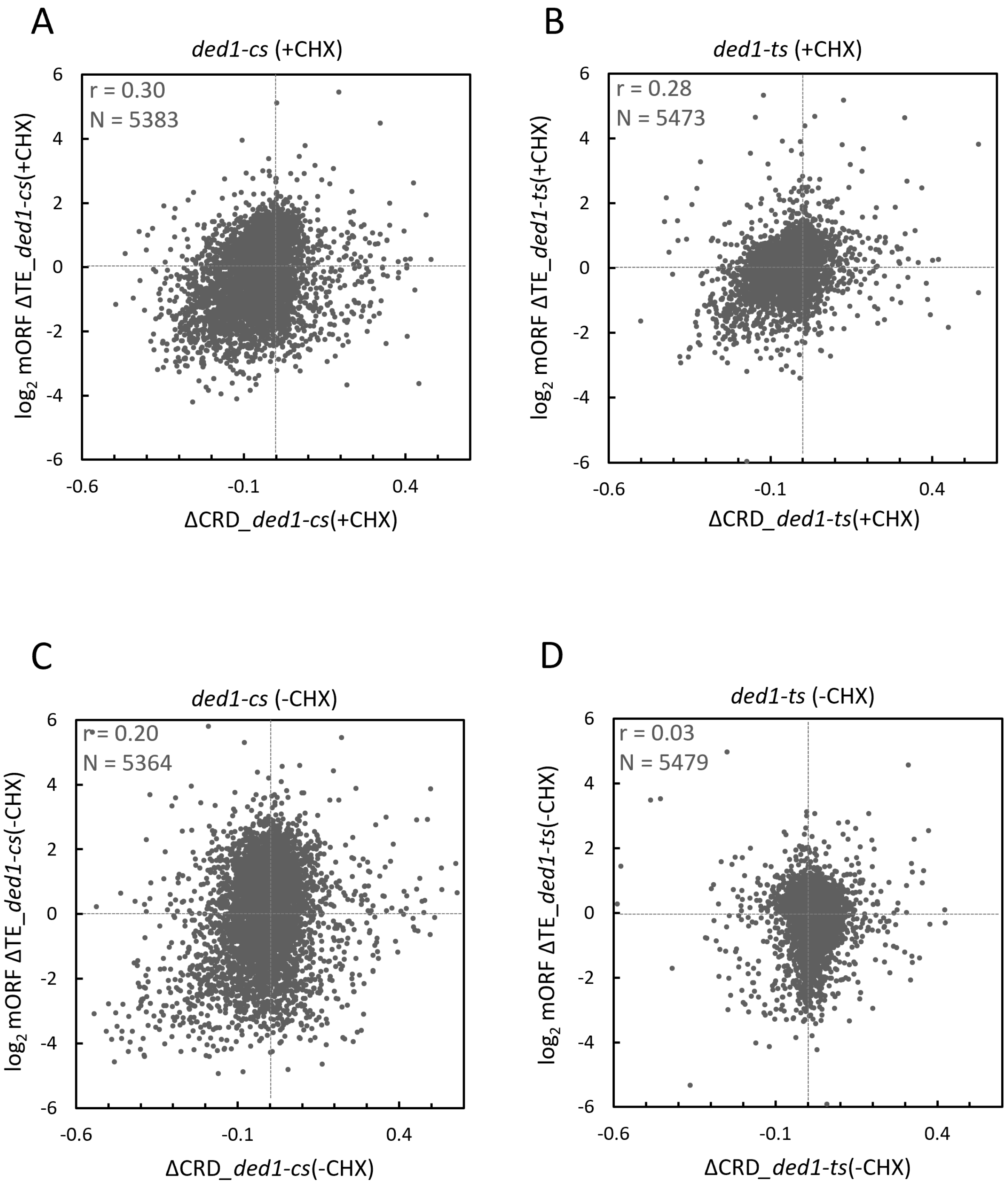

Figure S4. Cycloheximide pre-treatment of cells increases positive correlations between changes in TE and changes in center of ribosome density in *ded1* mutants vs. *DED1* cells. (A-D) Scatterplots comparing changes in TE and changes in center of ribosome density (CRD) for all

bioRxiv preprint doi: https://doi.org/10.64898/2026.04.10.717766; this version posted April 11, 2026. The copyright holder for this preprint (which was not certified by peer review) is the author/funder. This article is a US Government work. It is not subject to copyright under 17 USC 105 and is also made available for use under a CC0 license.

expressed genes in *ded1* mutant versus *DED1* cells for (A) *ded1-cs*(+CHX) (B) *ded1-ts*(+CHX) (C) *ded1-cs*(-CHX) and (D) *ded1-ts*(-CHX) experiments. Total number of genes in each comparison (N) and Pearson correlation coefficients (r) values are shown.

bioRxiv preprint doi: https://doi.org/10.64898/2026.04.10.717766; this version posted April 11, 2026. The copyright holder for this preprint (which was not certified by peer review) is the author/funder. This article is a US Government work. It is not subject to copyright under 17 USC 105 and is also made available for use under a CC0 license.

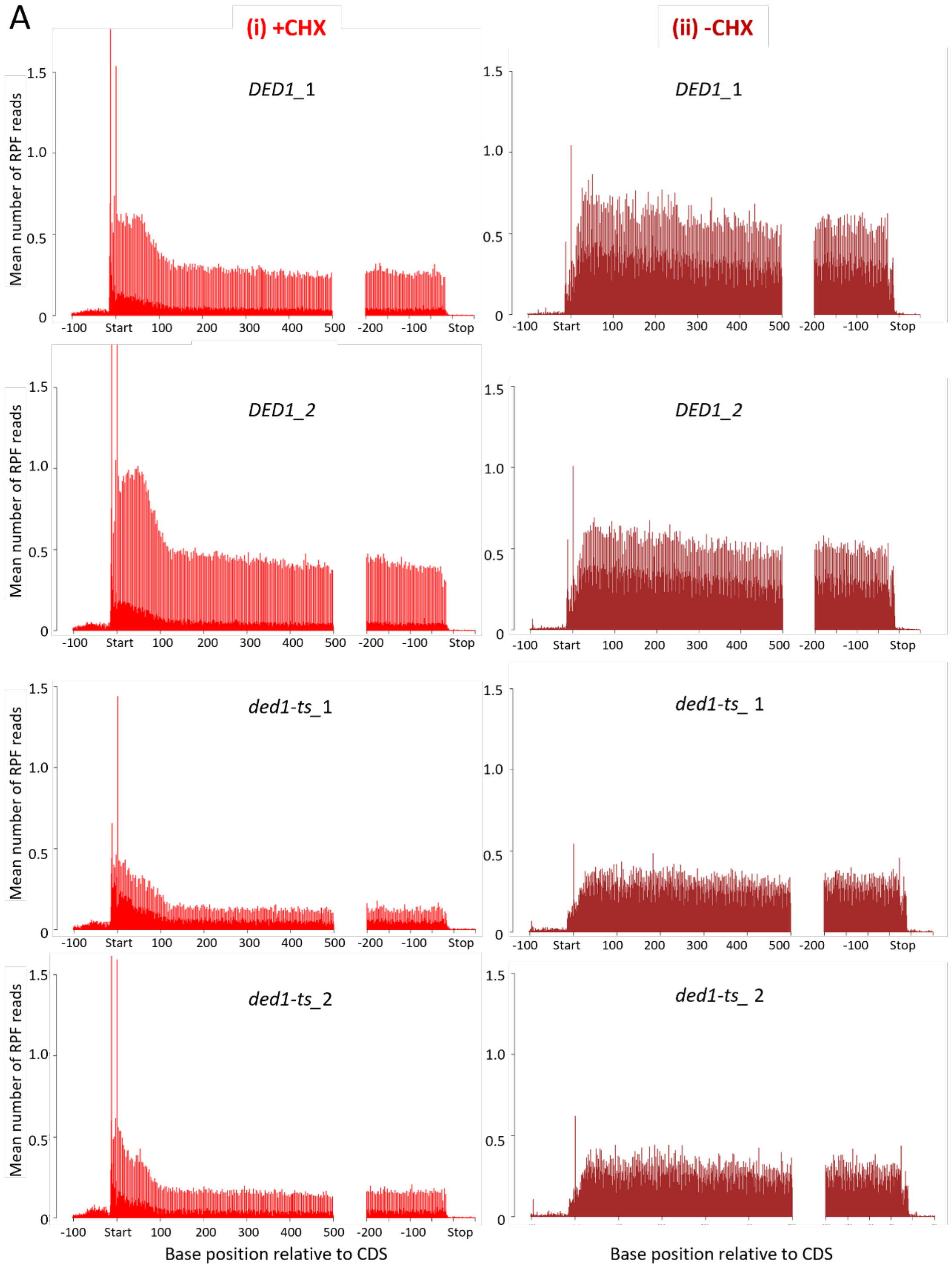

bioRxiv preprint doi: https://doi.org/10.64898/2026.04.10.717766; this version posted April 11, 2026. The copyright holder for this preprint (which was not certified by peer review) is the author/funder. This article is a US Government work. It is not subject to copyright under 17 USC 105 and is also made available for use under a CC0 license.

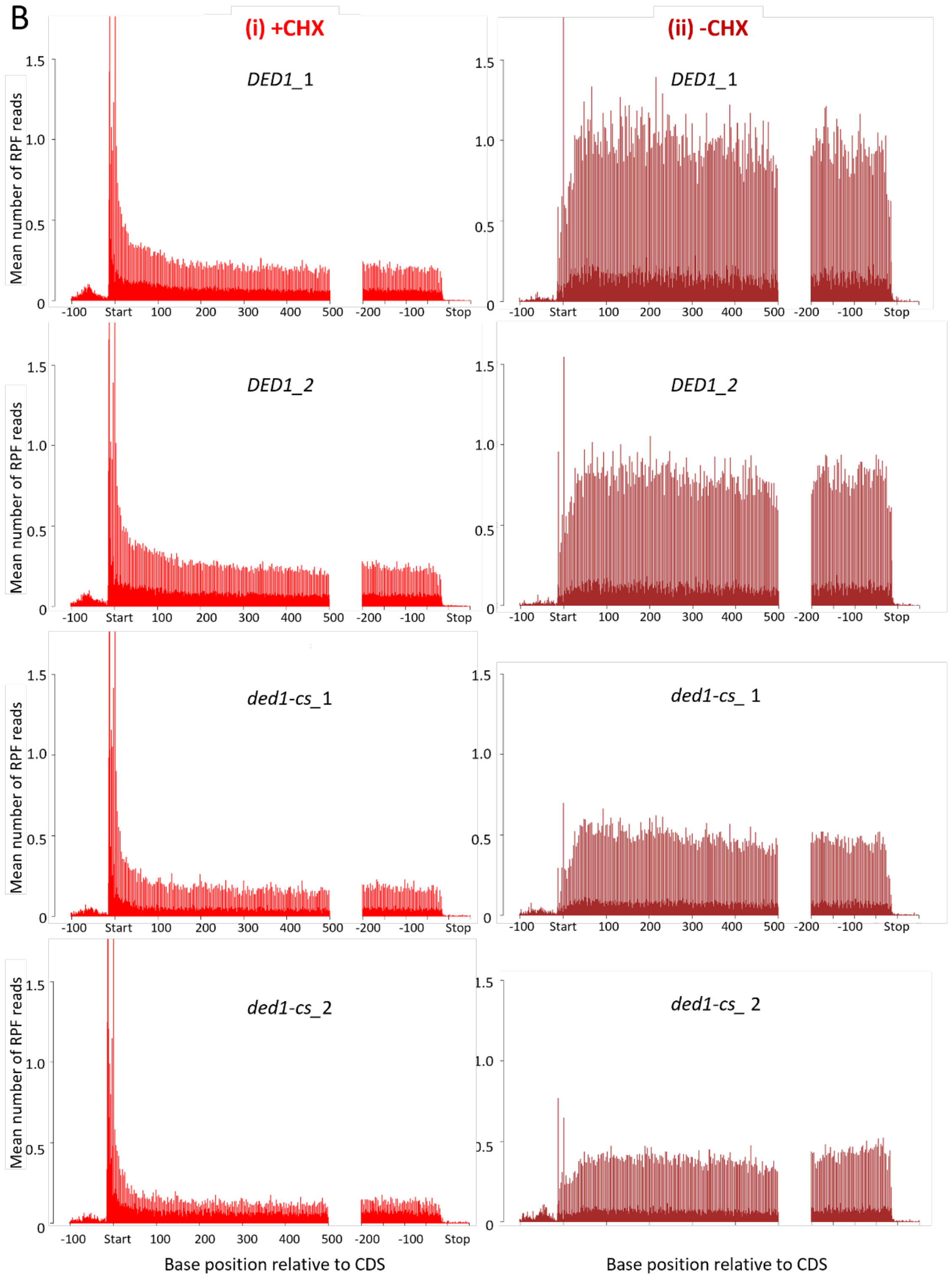

Figure S5. Pre-treating cells with cycloheximide increases RPF densities in the beginnings of mORFs throughout the translatome. Metagene plots of the mean number of RPF reads averaged

bioRxiv preprint doi: https://doi.org/10.64898/2026.04.10.717766; this version posted April 11, 2026. The copyright holder for this preprint (which was not certified by peer review) is the author/funder. This article is a US Government work. It is not subject to copyright under 17 USC 105 and is also made available for use under a CC0 license.

over all expressed genes at each nucleotide from bases -100 to -1 and 1 to 500 relative to the ATG start codon (position 1, “Start”) and from bases -200 to -1 and 50 bases downstream of the stop codon (position 1, “Stop”) calculated from Ribo-Seq data for two biological replicates (_1, _2) of *DED1* and *ded1-ts* strains (A) or *DED1* and *ded1-cs* strains (B) cultured at the non-permissive temperature for the corresponding *ded1* mutant, with cells pre-treated with CHX ((i), +CHX) or with CHX added only to cell lysates ((ii), -CHX).

bioRxiv preprint doi: https://doi.org/10.64898/2026.04.10.717766; this version posted April 11, 2026. The copyright holder for this preprint (which was not certified by peer review) is the author/funder. This article is a US Government work. It is not subject to copyright under 17 USC 105 and is also made available for use under a CC0 license.

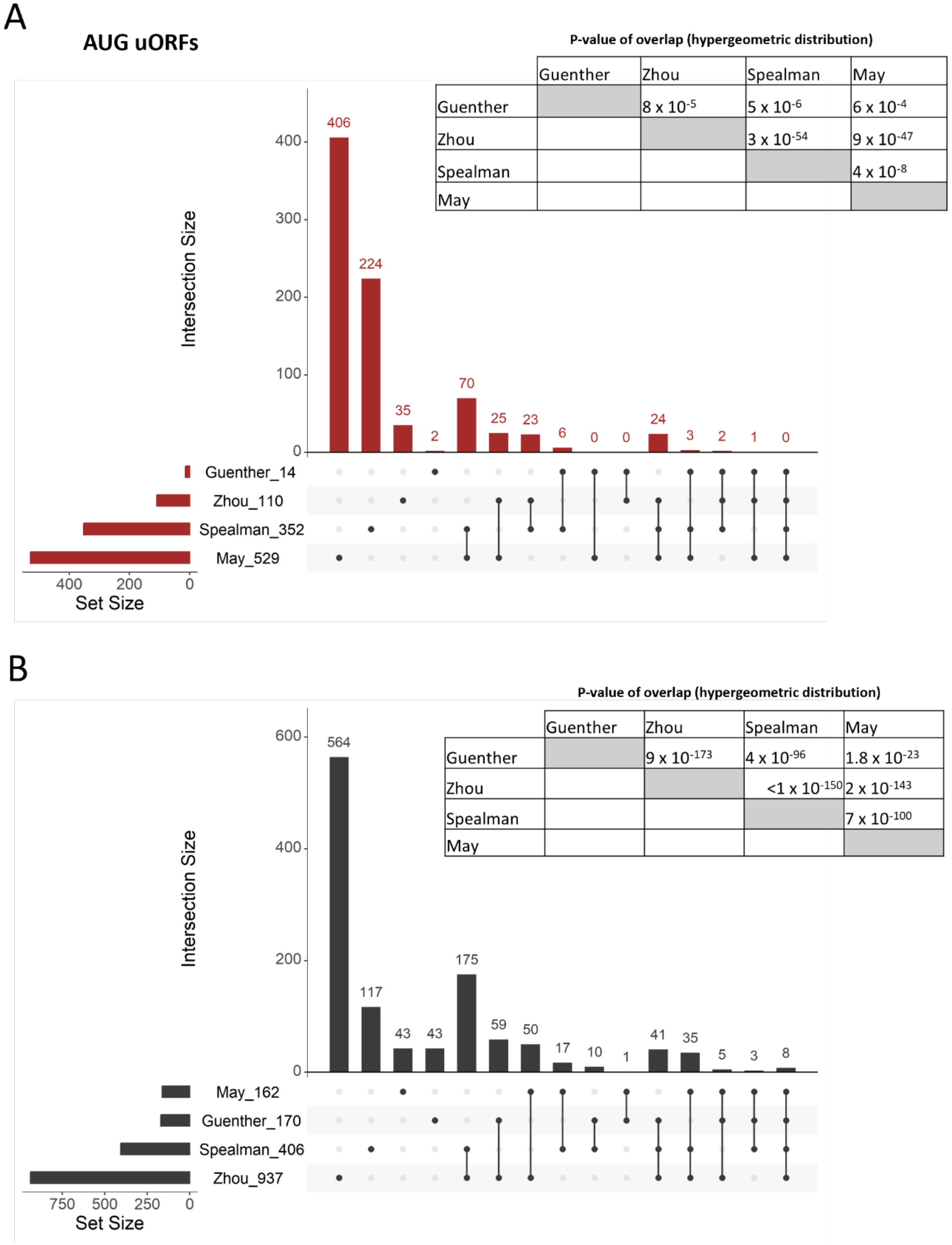

P-value of overlap (hypergeometric distribution) (A)

	Guenther	Zhou	Spealman	May
Guenther		8×10^{-5}	5×10^{-6}	6×10^{-4}
Zhou			3×10^{-54}	9×10^{-47}
Spealman				4×10^{-8}
May				

P-value of overlap (hypergeometric distribution) (B)

	Guenther	Zhou	Spealman	May
Guenther		9×10^{-173}	4×10^{-96}	1.8×10^{-23}
Zhou			$<1 \times 10^{-150}$	2×10^{-143}
Spealman				7×10^{-100}
May				

Figure S6. Highly significant overlaps among independent compilations of uORFs whose translation has been evaluated by Ribo-Seq analysis of *ded1* mutants. (A-B) Upset plots

bioRxiv preprint doi: https://doi.org/10.64898/2026.04.10.717766; this version posted April 11, 2026. The copyright holder for this preprint (which was not certified by peer review) is the author/funder. This article is a US Government work. It is not subject to copyright under 17 USC 105 and is also made available for use under a CC0 license.

depicting overlaps between sets of 14, 110, 352, and 529 AUG-initiated uORFs (A) or 162, 170, 406, and 937 NCC uORFs (B) identified by Guenther et al. (2), Zhou et al. (3), Spealman et al. (4), and May et al. (5), respectively. The first four columns list the numbers of uORFs unique to each set, while the last 11 columns depict the numbers of uORFs shared among 2, 3, or all 4 sets connected by lines. The significance of overlaps is indicated by the insets tabulating the P-values determined for all six pairwise comparisons using the hypergeometric distribution, based on 3156 total AUG uORFs and 74353 NCC uORFs identified in the sequences of annotated 5'UTRs for all yeast genes.

bioRxiv preprint doi: https://doi.org/10.64898/2026.04.10.717766; this version posted April 11, 2026. The copyright holder for this preprint (which was not certified by peer review) is the author/funder. This article is a US Government work. It is not subject to copyright under 17 USC 105 and is also made available for use under a CC0 license.

A

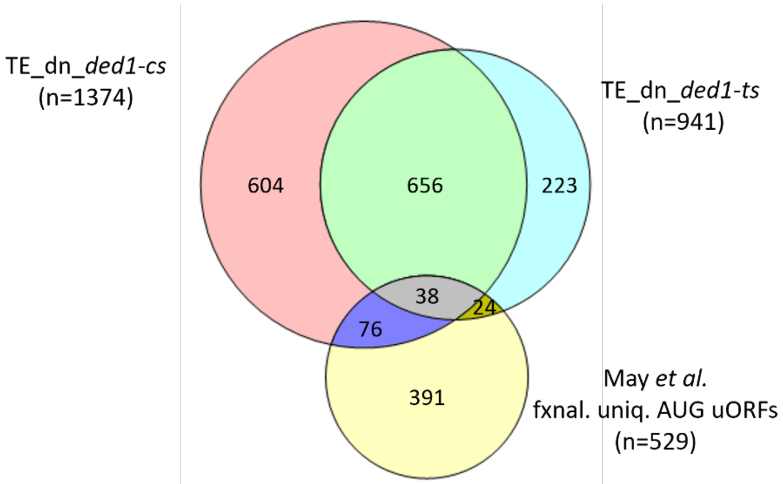

- TE_dn_*ded1-cs* ∩ fxnal. AUG uORFs: 1.1-fold depleted, P = 0.08
- TE_dn_*ded1-ts* ∩ fxnal. AUG uORFs: 1.4-fold depleted, P = 7 x 10^{-4}

B

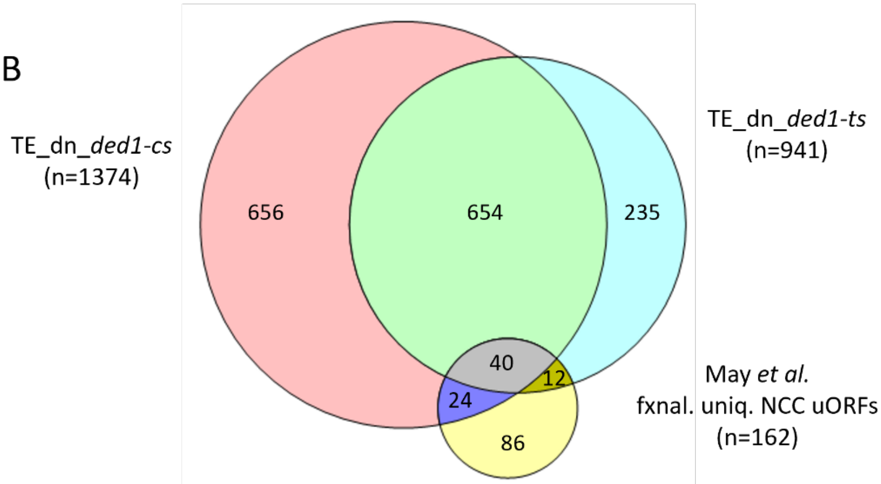

- TE_dn_*ded1-cs* ∩ fxnal. NCC uORFs: 1.6-fold enriched, P = 7 x 10^{-6}
- TE_dn_*ded1-ts* ∩ fxnal. NCC uORFs: 1.9-fold enriched, P =5 x 10^{-7}

C

Spealmannn *et al.* uORFs:
(i) TE_dn_*ded1-cs* ∩ conserved AUG uORFs: 1.1-fold enriched, P = 0.31
(ii) TE_dn_*ded1-ts* ∩ conserved AUG uORFs: 1.1-fold enriched, P= 0.30
(iii) TE_dn_*ded1-cs* ∩ conserved NCC uORFs: 2-fold enriched P = 2 x 10^{-27}
(iv) TE_dn_*ded1-ts* ∩ conserved NCC uORFs: 2.6-fold enriched P = 4 x 10^{-38}

Zhou *et al.* uORFs:
(v) TE_dn_*ded1-cs* ∩ translated AUG uORFs: 1.2-fold over-enriched P = 0.18
(vi) TE_dn_*ded1-ts* ∩ translated AUG uORFs: 1.2-fold over-enriched P =0.27
(vii) TE_dn_*ded1-cs* ∩ translated NCC uORFs: 1.8-fold enriched P = 7 x 10^{-49}
(vIII) TE_dn_*ded1-ts* ∩ translated NCC uORFs: 2.2-fold enriched P = 3 x 10^{-59}

Figure S7. Ded1-hyperdependent mRNAs are not significantly enriched for mRNAs with AUG-initiated uORFs. (A-B) Venn diagram showing overlap between Ded1-hyperdependent

bioRxiv preprint doi: https://doi.org/10.64898/2026.04.10.717766; this version posted April 11, 2026. The copyright holder for this preprint (which was not certified by peer review) is the author/funder. This article is a US Government work. It is not subject to copyright under 17 USC 105 and is also made available for use under a CC0 license.

mRNAs identified in *ded1-cs* or *ded1-ts* cells in -CHX experiments (mRNAs exhibiting ≥1.5-fold decrease in TE at FDR<0.05 in *ded1* mutant versus *DED1* cells, reported in Fig. S1C-D) and either 529 mRNAs containing functional AUG uORFs (A) or 162 mRNAs containing functional NCC uORFs (B) that were compiled by May et al. (5). These mRNA groups omit mRNAs that contain both AUG and NCC uORFs but include mRNAs that contain multiple AUG uORFs or multiple NCC uORFs, and thus contain mRNAs that uniquely harbor AUG or NCC functional uORFs (fxnal. uniq. AUG or NCC uORFs). **(C)** Same as (A-B) except analyzing evolutionarily conserved uniquely AUG (n=352) or uniquely NCC (n=406) uORFs identified by Spealmann et al. (4), or analyzing translated uniquely AUG (n=110) or uniquely NCC (n=937) uORFs identified by Zhou et al. (3).

bioRxiv preprint doi: https://doi.org/10.64898/2026.04.10.717766; this version posted April 11, 2026. The copyright holder for this preprint (which was not certified by peer review) is the author/funder. This article is a US Government work. It is not subject to copyright under 17 USC 105 and is also made available for use under a CC0 license.

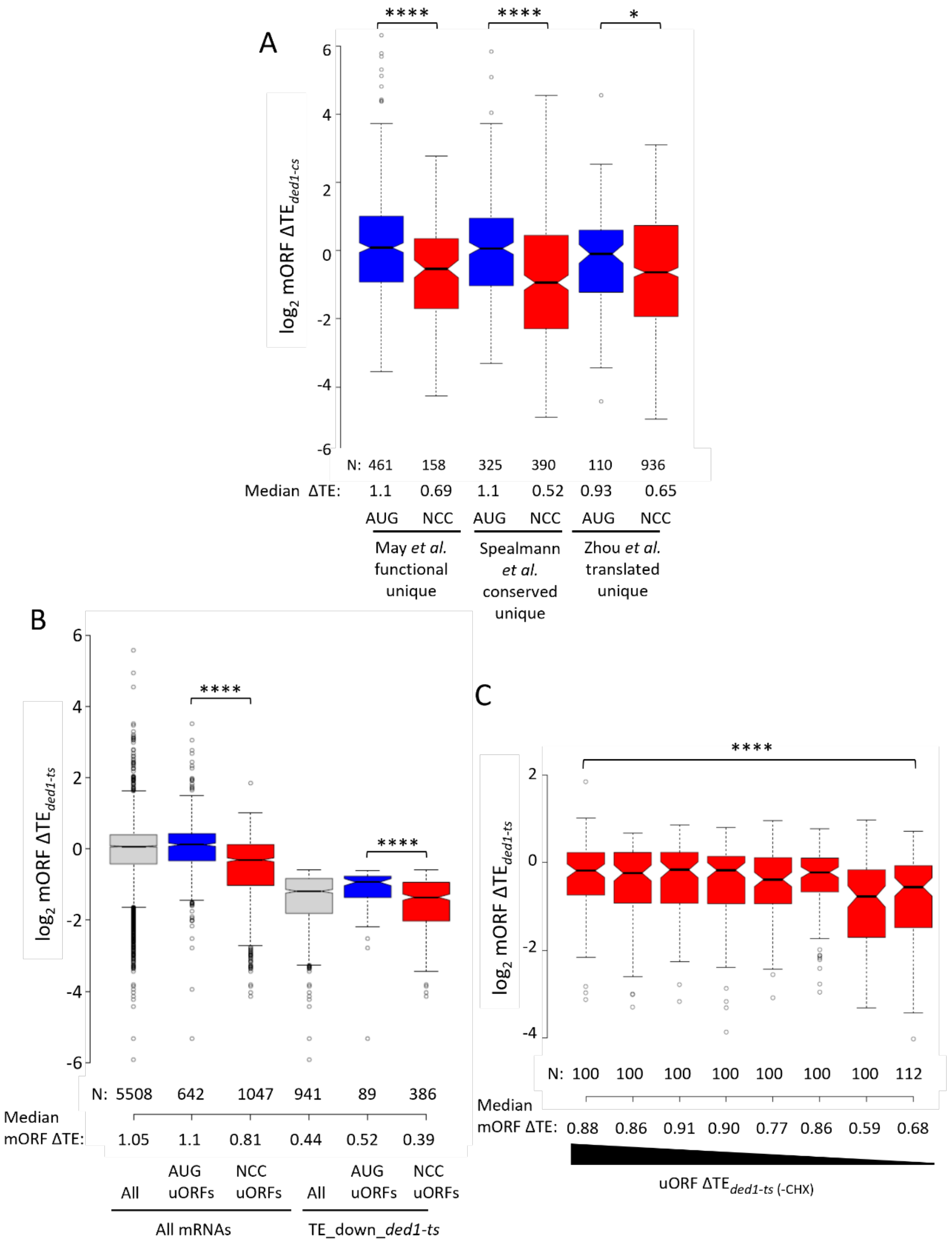

bioRxiv preprint doi: https://doi.org/10.64898/2026.04.10.717766; this version posted April 11, 2026. The copyright holder for this preprint (which was not certified by peer review) is the author/funder. This article is a US Government work. It is not subject to copyright under 17 USC 105 and is also made available for use under a CC0 license.

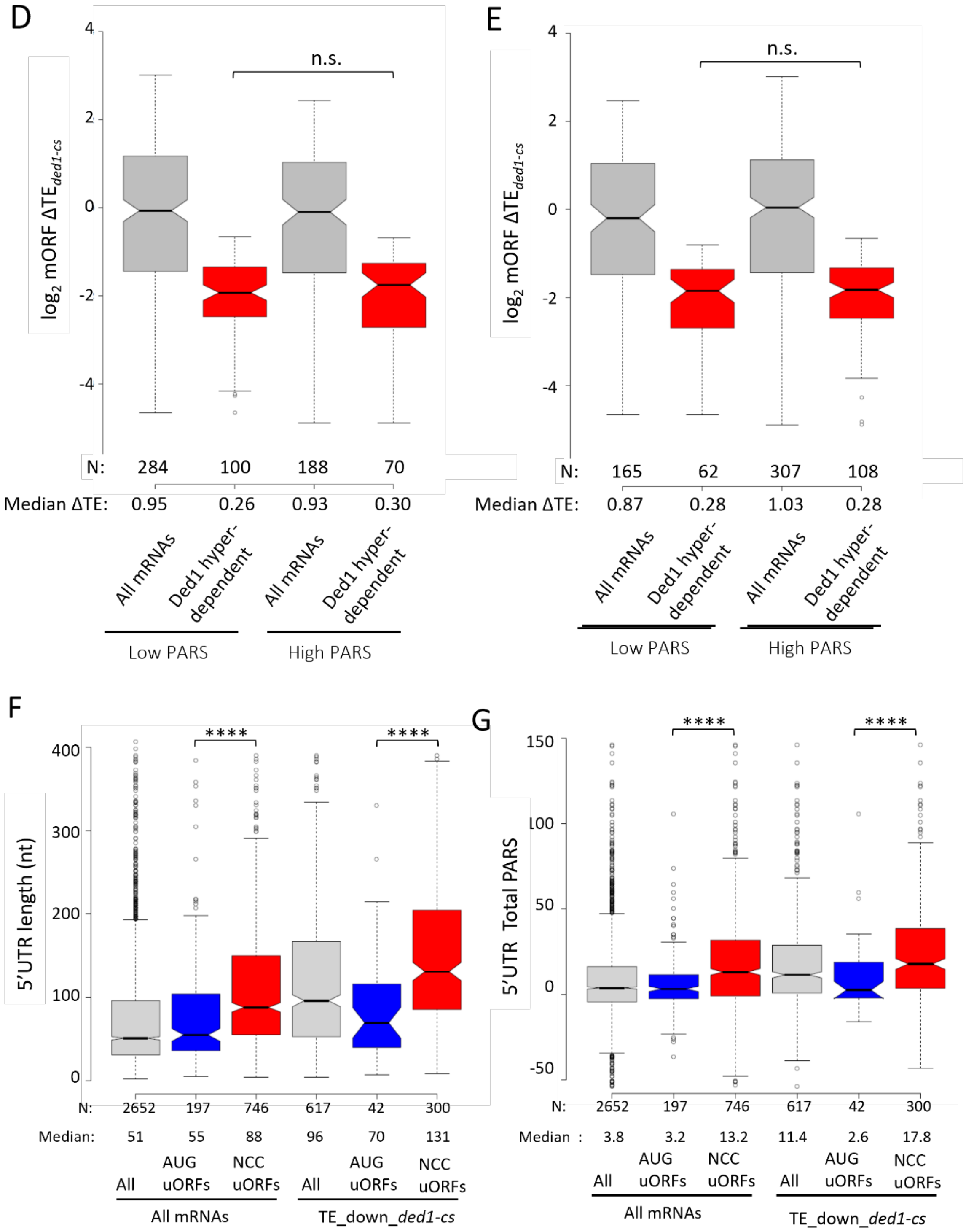

Figure S8. Presence of NCC-initiated uORFs is associated with, but not functionally linked to, heightened dependence on Ded1 for translation of mORFs. (A) Notched box plots

bioRxiv preprint doi: https://doi.org/10.64898/2026.04.10.717766; this version posted April 11, 2026. The copyright holder for this preprint (which was not certified by peer review) is the author/funder. This article is a US Government work. It is not subject to copyright under 17 USC 105 and is also made available for use under a CC0 license.

comparing $\log_2$ fold-changes in mORF TE observed in *ded1-cs* versus *DED1* cells from -CHX cultures for mRNAs containing either unique AUG uORFs (cols. 1,3 & 5) or NCC uORFs (cols. 2,4 & 6) compiled by May et al. (5), (cols. 1-2), Spealmann et al. (4) (cols. 3-4), or Zhou et al. (3) (cols. 5-6) and described in Fig. S5A-C. **(B-C) Ded1-hyperdependence conferred by the *ded1-ts* mutation is largely independent of increased AUG- or NCC-initiated uORF translation.** Analysis identical to Fig. 3A-B conducted using *ded1-ts* (-CHX) Ribo-Seq data. **(D-E) Higher propensity for secondary structures downstream of NCC uORFs does not confer heightened dependence on Ded1 for translation of mORFs.** Similar to Fig. 3C but summing up the PARS scores for nucleotides 16-30 (A) or 16-60 (B) downstream of the start codon of the NCC uORFs. **(F-G) Longer and structure prone 5'UTRs of mRNAs containing NCC uORFs confer heightened dependence on Ded1 for translation of mORFs.** Notched block blots of the distributions of 5'UTR lengths (A) or Total PARS scores (B) for the same groups of mRNAs analyzed in Fig. 3A for which data was reported by Kertesz et al. (6). Results of Mann-Whitney U-tests for all panels are summarized as: ****, $P<0.0001$; ***, $P<0.001$; n.s., not significant, $P>0.05$.

bioRxiv preprint doi: https://doi.org/10.64898/2026.04.10.717766; this version posted April 11, 2026. The copyright holder for this preprint (which was not certified by peer review) is the author/funder. This article is a US Government work. It is not subject to copyright under 17 USC 105 and is also made available for use under a CC0 license.

A

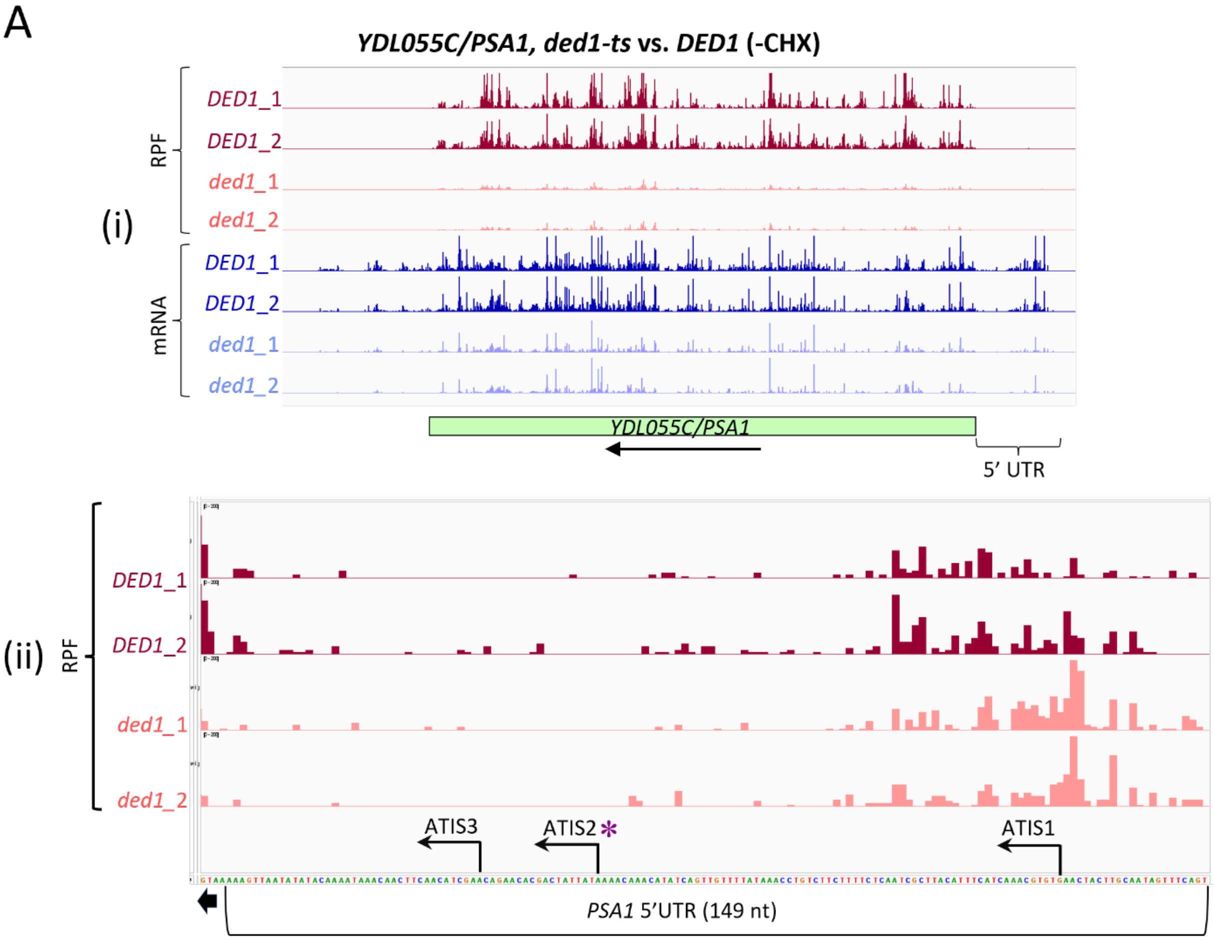

(iii)

	ΔRPF	ΔmRNA	ΔTE	ΔTE (FDR)	ΔTE (P val.)
mORF	0.18	0.38	0.47	3.3 x 10^{-9}	2.3 x 10^{-10}
ATIS1	1.3	0.34	3.7	1.3 x 10^{-13}	3.3 x 10^{-15}
ATIS2	0.44	0.34	1.3	0.64	0.52
ATIS3	0.68	0.34	2.0	0.16	0.08

bioRxiv preprint doi: https://doi.org/10.64898/2026.04.10.717766; this version posted April 11, 2026. The copyright holder for this preprint (which was not certified by peer review) is the author/funder. This article is a US Government work. It is not subject to copyright under 17 USC 105 and is also made available for use under a CC0 license.

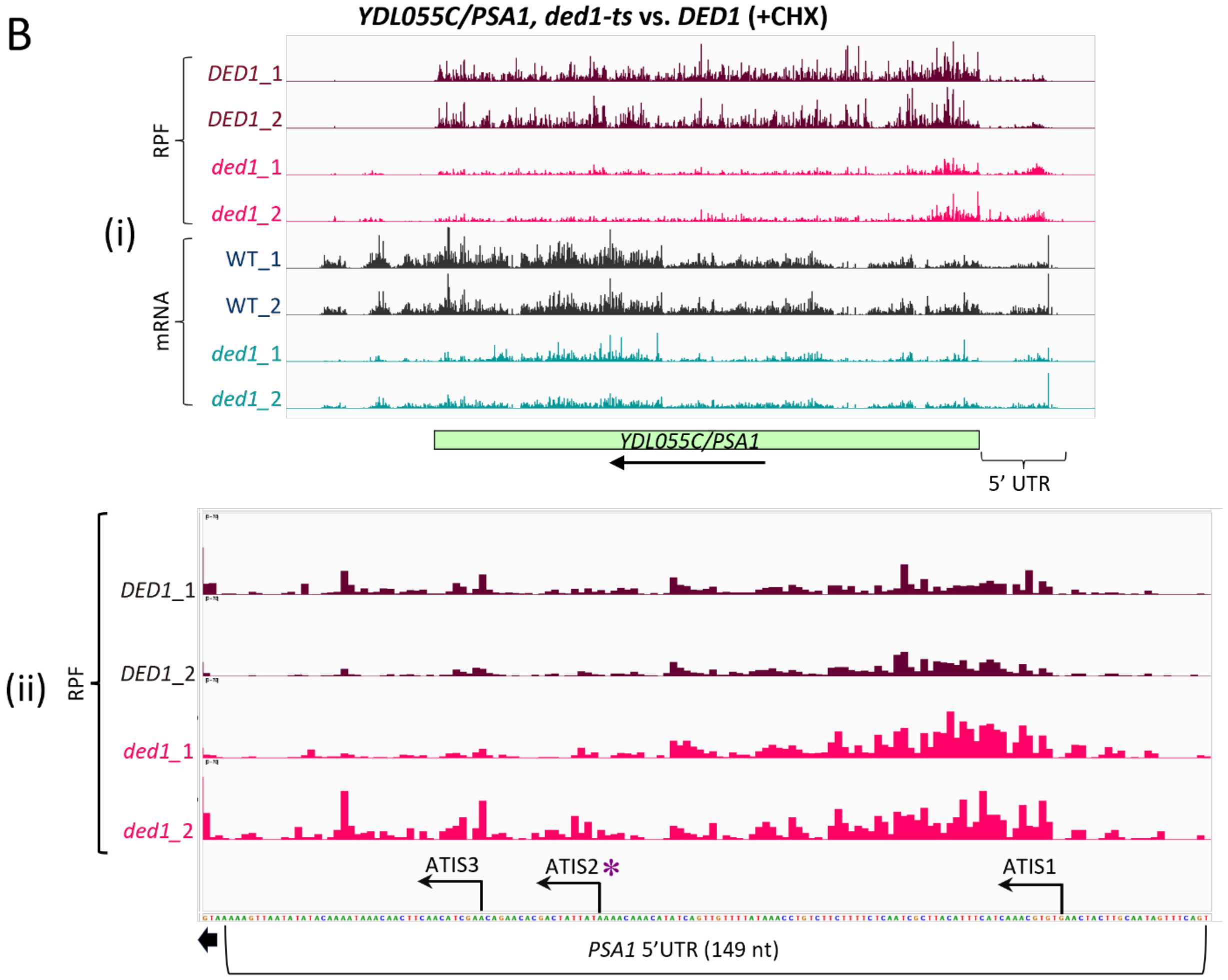

(iii)

	ΔRPF	ΔmRNA	ΔTE	ΔTE (FDR)	ΔTE (P val.)
mORF	0.2	0.37	0.54	0.04	0.005
ATIS1	1.4	0.34	4.1	6.3×10^{-6}	2.8×10^{-7}
ATIS2	1.0	0.34	3.0	0.05	0.015
ATIS3	0.96	0.34	2.9	0.08	0.03

bioRxiv preprint doi: https://doi.org/10.64898/2026.04.10.717766; this version posted April 11, 2026. The copyright holder for this preprint (which was not certified by peer review) is the author/funder. This article is a US Government work. It is not subject to copyright under 17 USC 105 and is also made available for use under a CC0 license.

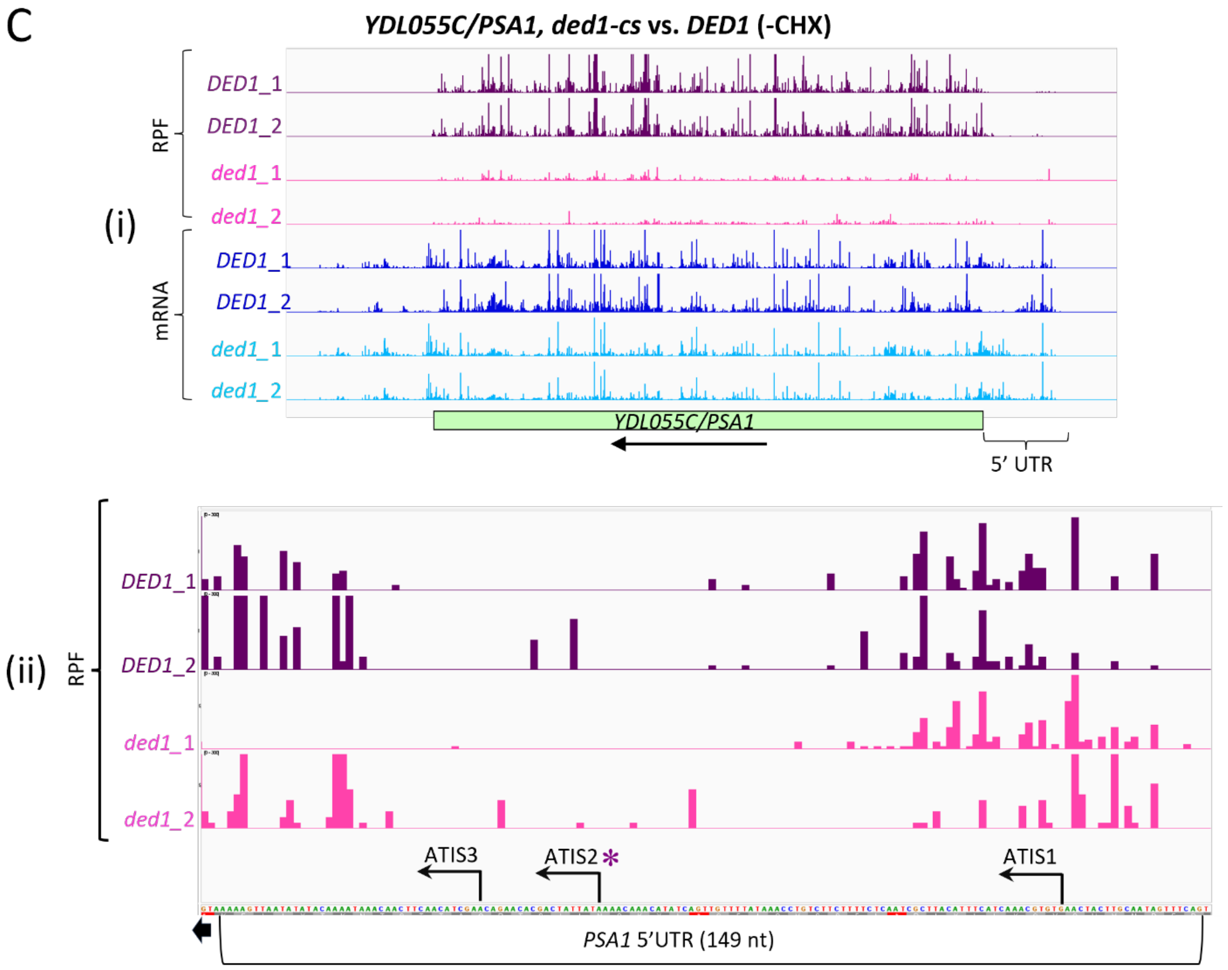

(iii)

	ΔRPF	ΔmRNA	ΔTE	ΔTE (FDR)	ΔTE (P val.)
mORF	0.27	0.39	0.69	0.44	0.35
ATIS1	1.4	0.38	3.8	0.09	0.058
ATIS2	1.3	0.38	3.4	0.45	0.37
ATIS3	1.4	0.38	3.7	0.41	0.33

bioRxiv preprint doi: https://doi.org/10.64898/2026.04.10.717766; this version posted April 11, 2026. The copyright holder for this preprint (which was not certified by peer review) is the author/funder. This article is a US Government work. It is not subject to copyright under 17 USC 105 and is also made available for use under a CC0 license.

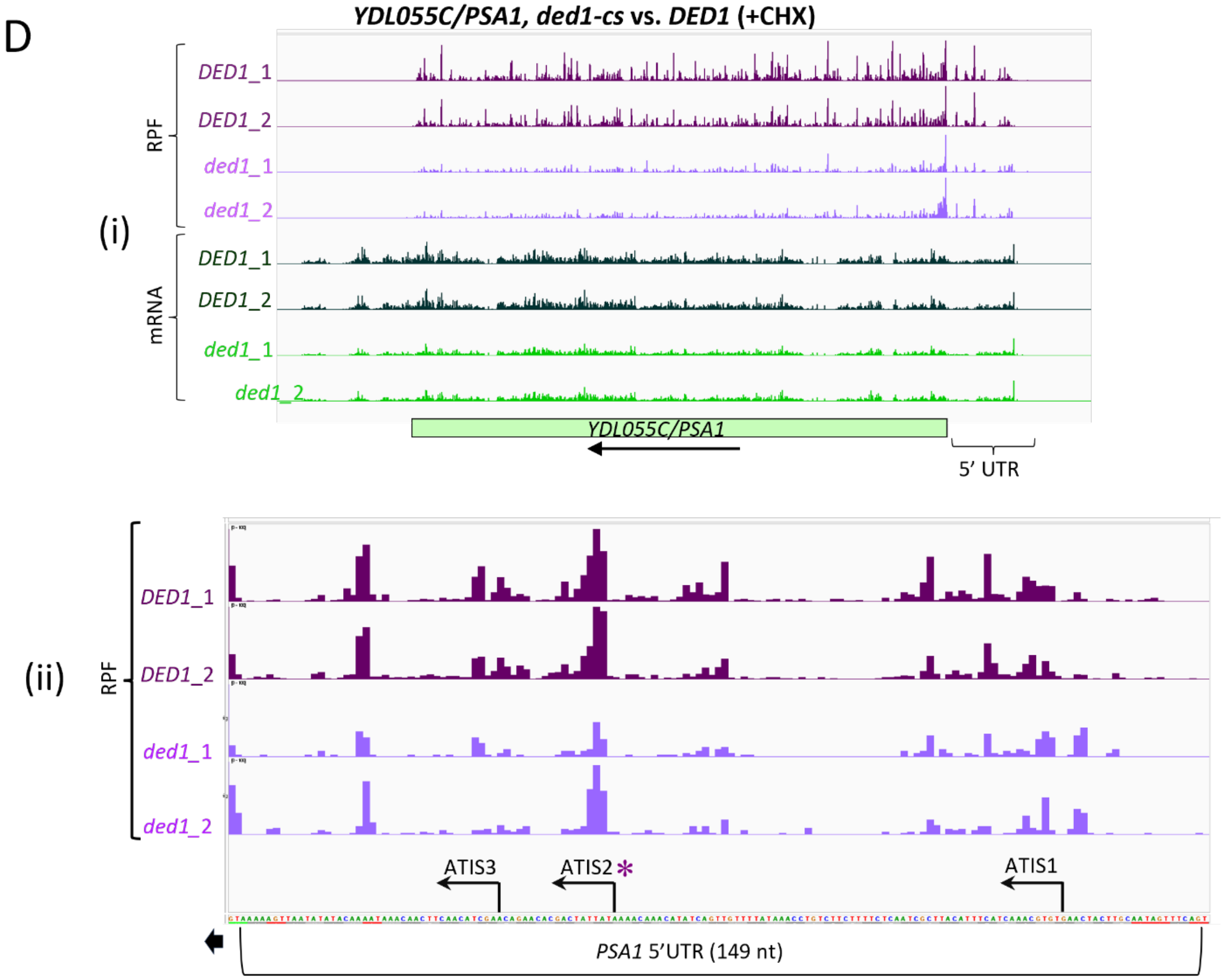

(iii)

	ΔRPF	ΔmRNA	ΔTE	ΔTE (FDR)	ΔTE (P val.)
mORF	0.41	0.75	0.56	1.7×10^{-5}	6.2×10^{-6}
ATIS1	0.78	0.70	1.1	0.65	0.59
ATIS2	0.70	0.70	1.0	0.99	0.99
ATIS3	0.70	0.70	1.0	0.99	0.98

Figure S9. *ded1* mutations do not confer increased translation of ATIS2 associated with decreased translation of the *PSA1* mORF in Ribo-Seq conducted without CHX-treatment of cells. (A)-(D). Panels (i)-(ii) Gene-browser depictions of RPF and mRNA counts in the 5'UTR and mORF (i) or only the 5'UTR (ii) of *PSA1* determined by Ribo-Seq analysis of two replicates (_1, _2) of *DED1* and *ded1-ts* cells (A-B) or *DED1* and *ded1-cs* cells (C-D) prepared with CHX

bioRxiv preprint doi: https://doi.org/10.64898/2026.04.10.717766; this version posted April 11, 2026. The copyright holder for this preprint (which was not certified by peer review) is the author/funder. This article is a US Government work. It is not subject to copyright under 17 USC 105 and is also made available for use under a CC0 license.

treatment of either lysates (-CHX, panels A & C) or cells (+CHX, panels B & D). The GUG, AUA, and AAG start codons of three NCC ATISs (dubbed 1, 2, and 3) are indicated above the 149 nt 5'UTR sequence of *PSA1* in (ii), with the asterisk indicating the importance of ATIS2 in Ded1-mediated enhanced translation of the *PSA1* mORF, as reported by Guenther et al. (2). All eight tracks in panel (i) or four tracks in panel (ii) have the same ranges of read counts. **(iii)** Tabulated changes in RPFs, mRNA, and TEs, with FDR and P-values for the ΔTE values, determined by DESeq2 analysis, conferred by the *ded1* mutation and condition of Ribo-Seq analysis (-CHX vs. +CHX) indicated at the top of the figure. Statistically significant TE changes ($P < 0.05$) are indicated with light green shading of the relevant cells. Whereas the mORF TE is reduced by the *ded1* mutation in three of the four datasets (panels A, B & D), an inversely correlated TE increase for ATIS2, in the manner predicted by the START model, was observed only for the *ded1-ts* mutation in the +CHX dataset (panel B). Slightly different ΔmRNA values for mORFs versus ATISs 1-3 in each panel result from differences in the number of entries in the fpcount input files subjected to two separate DESeq2 analyses of TE changes for uORFs or mORFs. For the uORF TE analyses, the fpcount file had 8962 entries comprised of 3315 uORFs and 5647 CDSs, whereas only the 5647 CDS entries comprised the fpcount file used for mORF TE analysis. We included the CDS entries with the uORF entries for the uORF TE analysis to reduce imprecision resulting from generally low read counts for the short uORFs compared to the much higher read counts for the longer mORFs encoded within the same transcripts, as done previously (7).

bioRxiv preprint doi: https://doi.org/10.64898/2026.04.10.717766; this version posted April 11, 2026. The copyright holder for this preprint (which was not certified by peer review) is the author/funder. This article is a US Government work. It is not subject to copyright under 17 USC 105 and is also made available for use under a CC0 license.

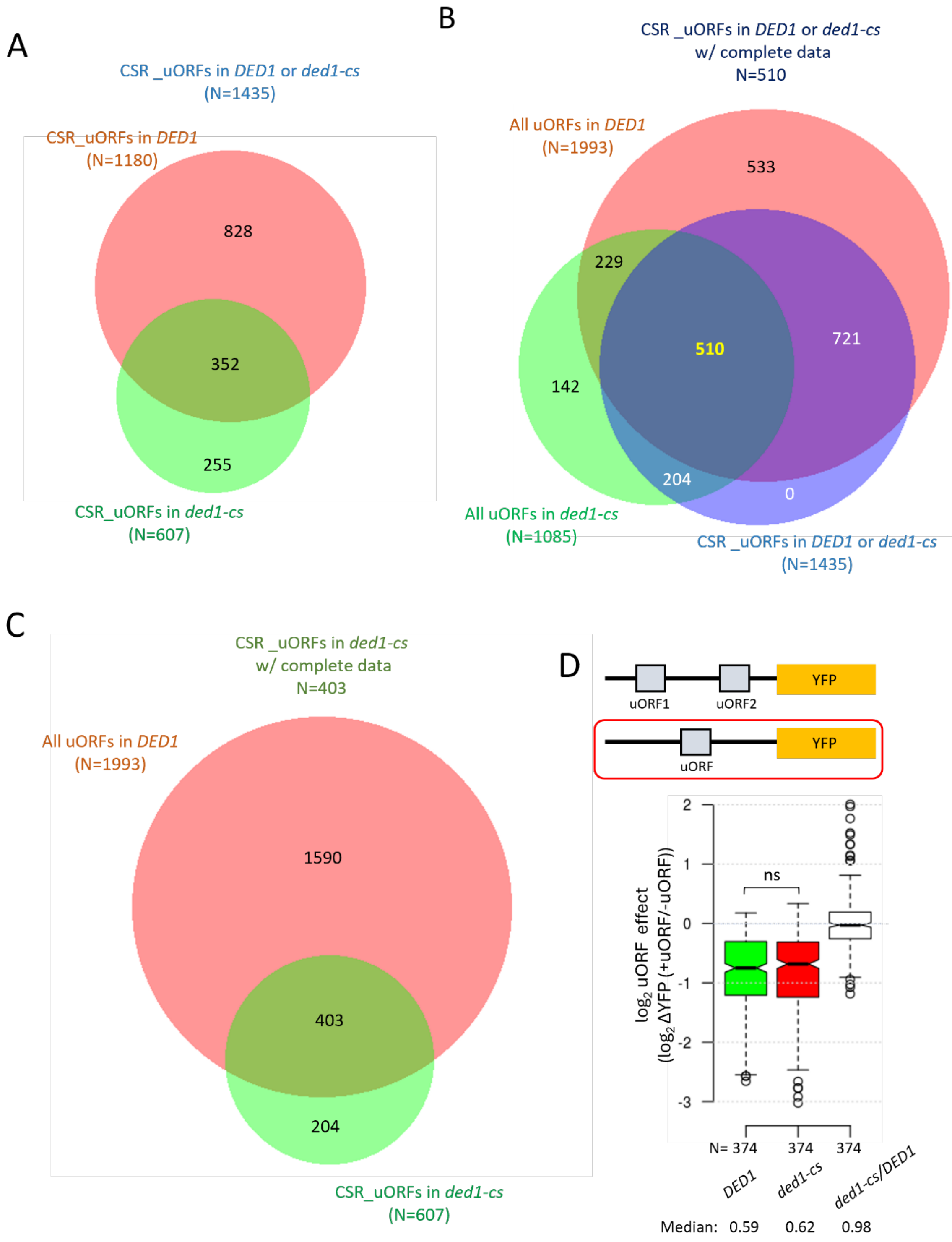

Figure S10. Compilation of results from MPRA of the FACS-uORF library in *ded1-cs* vs. *DED1* cells. (A) Venn diagram depicting overlap between the two sets of YFP reporters whose

bioRxiv preprint doi: https://doi.org/10.64898/2026.04.10.717766; this version posted April 11, 2026. The copyright holder for this preprint (which was not certified by peer review) is the author/funder. This article is a US Government work. It is not subject to copyright under 17 USC 105 and is also made available for use under a CC0 license.

uORFs were judged to CSR by comparing expression of replicates for the matched WT_uORF and Mut_uORF in the *DED1* strain (N=1180) or *ded1-cs* mutant (N=607). **(B)** Venn diagram depicting overlap between the set of 1435 reporters whose uORFs were judged to be CSR in either *DED1* or *ded1-cs* cells (union of two sets in (A), blue), the 1993 reporters for which replicate data were obtained for both WT_uORF and Mut_uORF reporters in *DED1* cells (red), and the 1085 reporters with replicate data for both WT_uORF and Mut_uORF reporters in *ded1-cs* cells (green). **(C)** Venn diagram depicting overlap between the set of 1993 reporters for which replicate data was obtained for both WT_uORF and Mut_uORF reporters in *DED1* cells (also analyzed in (B), red) and the 607 reporters whose uORFs were judged to CSR in *ded1-cs* cells (also analyzed in (A), green). **(D)** Analysis identical to that in Fig. 4B(ii) but for the subset of 374 CSR uORFs from the group of 510 described in panel C that contain only a single uORF that was mutated in the FACS-uORF library, as depicted with red outline in the schematic above.

bioRxiv preprint doi: https://doi.org/10.64898/2026.04.10.717766; this version posted April 11, 2026. The copyright holder for this preprint (which was not certified by peer review) is the author/funder. This article is a US Government work. It is not subject to copyright under 17 USC 105 and is also made available for use under a CC0 license.

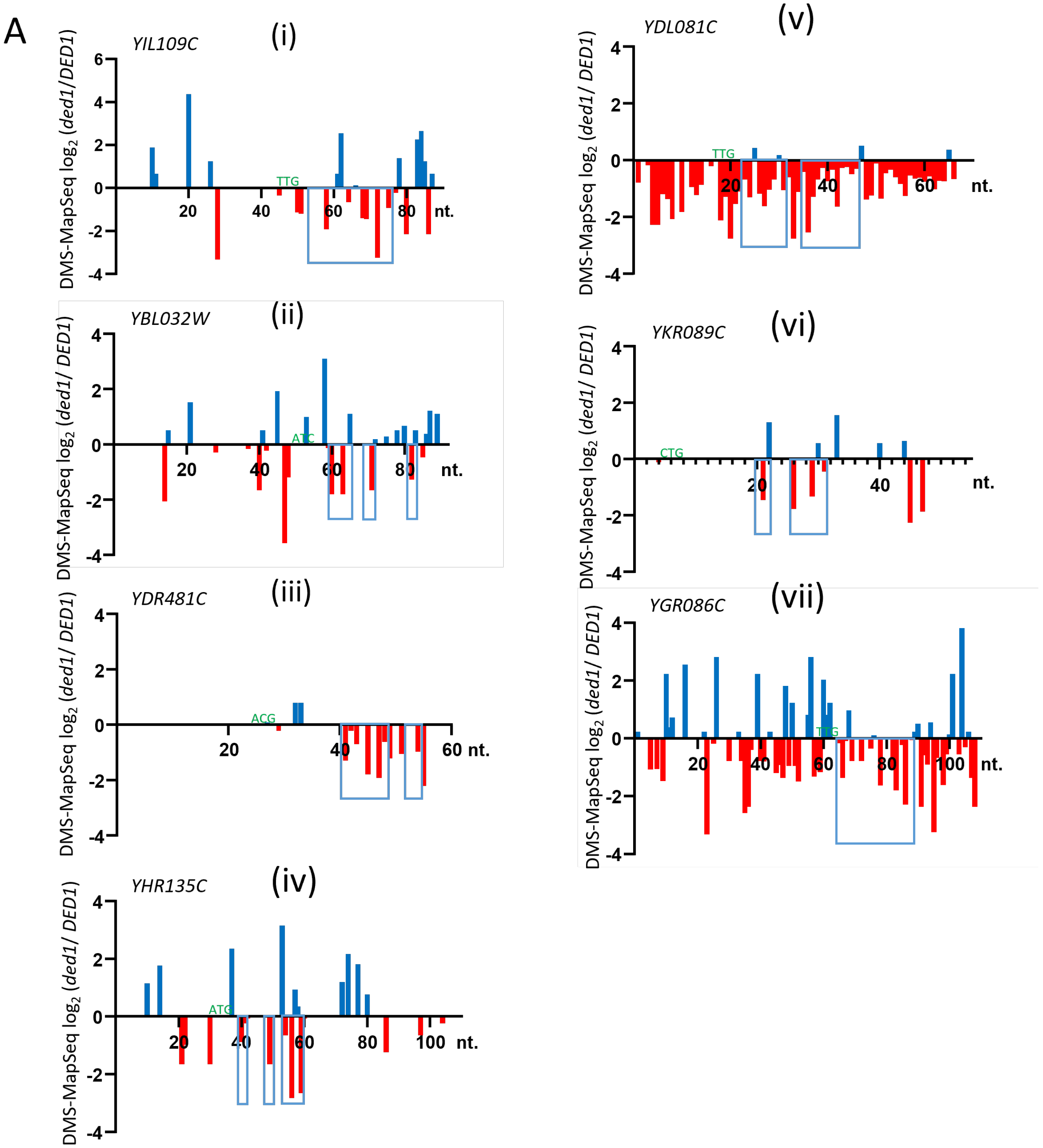

bioRxiv preprint doi: https://doi.org/10.64898/2026.04.10.717766; this version posted April 11, 2026. The copyright holder for this preprint (which was not certified by peer review) is the author/funder. This article is a US Government work. It is not subject to copyright under 17 USC 105 and is also made available for use under a CC0 license.

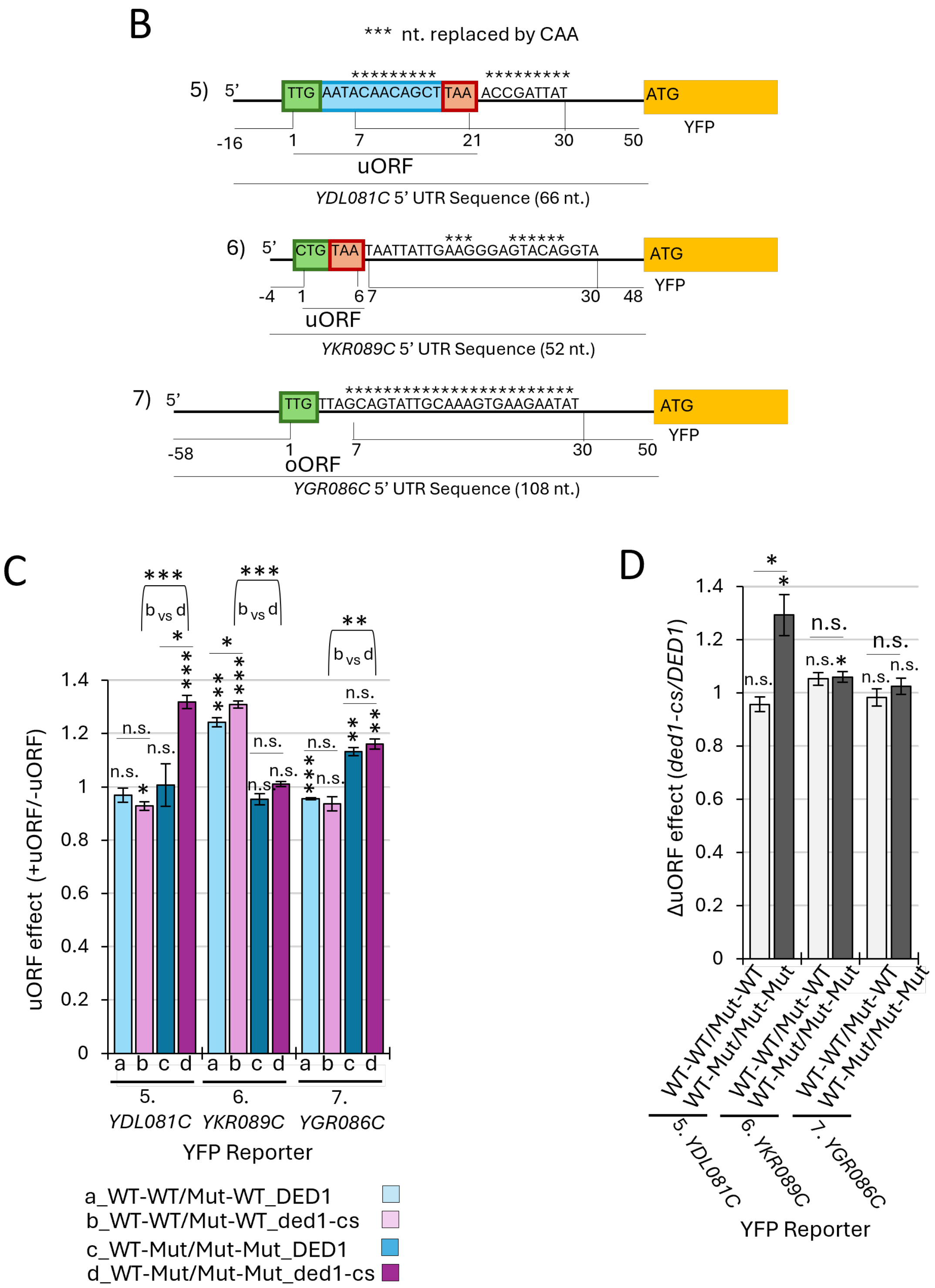

bioRxiv preprint doi: https://doi.org/10.64898/2026.04.10.717766; this version posted April 11, 2026. The copyright holder for this preprint (which was not certified by peer review) is the author/funder. This article is a US Government work. It is not subject to copyright under 17 USC 105 and is also made available for use under a CC0 license.

Figure S11. Reporter analyses indicating that uORFs at *YDL081C, YKR089C,* and *YGR086C* do not conform to the Ded1-START model. (A) Compilations of the published DMS-MapSeq data from Guenther et al. (2) presented as $\log_2$ ratios of read counts (normalized reverse transcription stops) plotted against nucleotide position (nt) in the 5'UTR (5' to 3' from the cap) obtained from *ded1-ts* mutant vs. *DED1* cells after 5 min at the non-permissive growth temperature for (i)-(iv) the four genes subjected to mutational analysis of uORFs and proximal downstream structures with results presented in Fig. 6, and (v)-(vii) the three genes similarly analyzed in panels B-D below. Negative values on y-axes (red bars) signify nucleotides more unwound (and DMS-modified) in *DED1* vs. *ded1-ts* cells. Violet boxes delineate regions downstream of the uORF start codons (green type on or above x-axis, not exactly to scale) that were subjected to CAA replacements of nucleotides unwound by Ded1 to disrupt structures 3'-proximal to the uORF start codons that are unwound by Ded1, which were separately substituted with AAG or AAA triplets to mutate the uORF start sites in the presence or absence of CAA substitutions. **(B)-(D)** Depiction of *YFP* reporter constructs (B) and analyses of reporter expression in *DED1* and *ded1-cs* cells for three uORFs at genes *YDL081C, YGR086C,* and *YKR089C* (C-D) presented exactly as described above for the four reporters examined in Figs. 6A, C & D, summarizing data from 3 biological replicates for each reporter/strain combination. Results for all three gene/uORFs analyzed here depart from one or more predictions of the Ded1-START model, as follows. The data in (C) for *YDL081C* and *YGR086C* reveal that their uORFs are not significantly more inhibitory in *ded1-cs* vs. *DED01* cells, exhibiting essentially the same uORF effects in the two strains, when the WT downstream structures are present (reporters 5 & 7, col. a vs. b); and eliminating the downstream structures unexpectedly renders the uORFs stimulatory, now exhibiting uORF effects >1, in both *DED1* and *ded1-cs* cells for *YGR086C* (reporter 7, col. c vs. a and col. d vs. b) or only in *ded1-cs*

bioRxiv preprint doi: https://doi.org/10.64898/2026.04.10.717766; this version posted April 11, 2026. The copyright holder for this preprint (which was not certified by peer review) is the author/funder. This article is a US Government work. It is not subject to copyright under 17 USC 105 and is also made available for use under a CC0 license.

cells for *YDL081C* (reporter 5, col. d vs. b). For *YKR089C,* the uORF appears to be stimulatory rather than inhibitory in both strains, conferring uORF effects >1 in the presence of the structures (reporter 6, col. a-b); and removing the downstream structure renders the uORF ineffectual, now exhibiting a uORF effect of ~1.0 (reporter 6, col. c vs. a and d vs. b). Consequently, the results in (D) show that none of the three reporters exhibit ΔuORF effect (*ded1-cs/DED1*) ratios significantly < 1.0 for the reporters with WT structures (light grey bars) but not those with mutated structures (adjacent black bars).

bioRxiv preprint doi: https://doi.org/10.64898/2026.04.10.717766; this version posted April 11, 2026. The copyright holder for this preprint (which was not certified by peer review) is the author/funder. This article is a US Government work. It is not subject to copyright under 17 USC 105 and is also made available for use under a CC0 license.

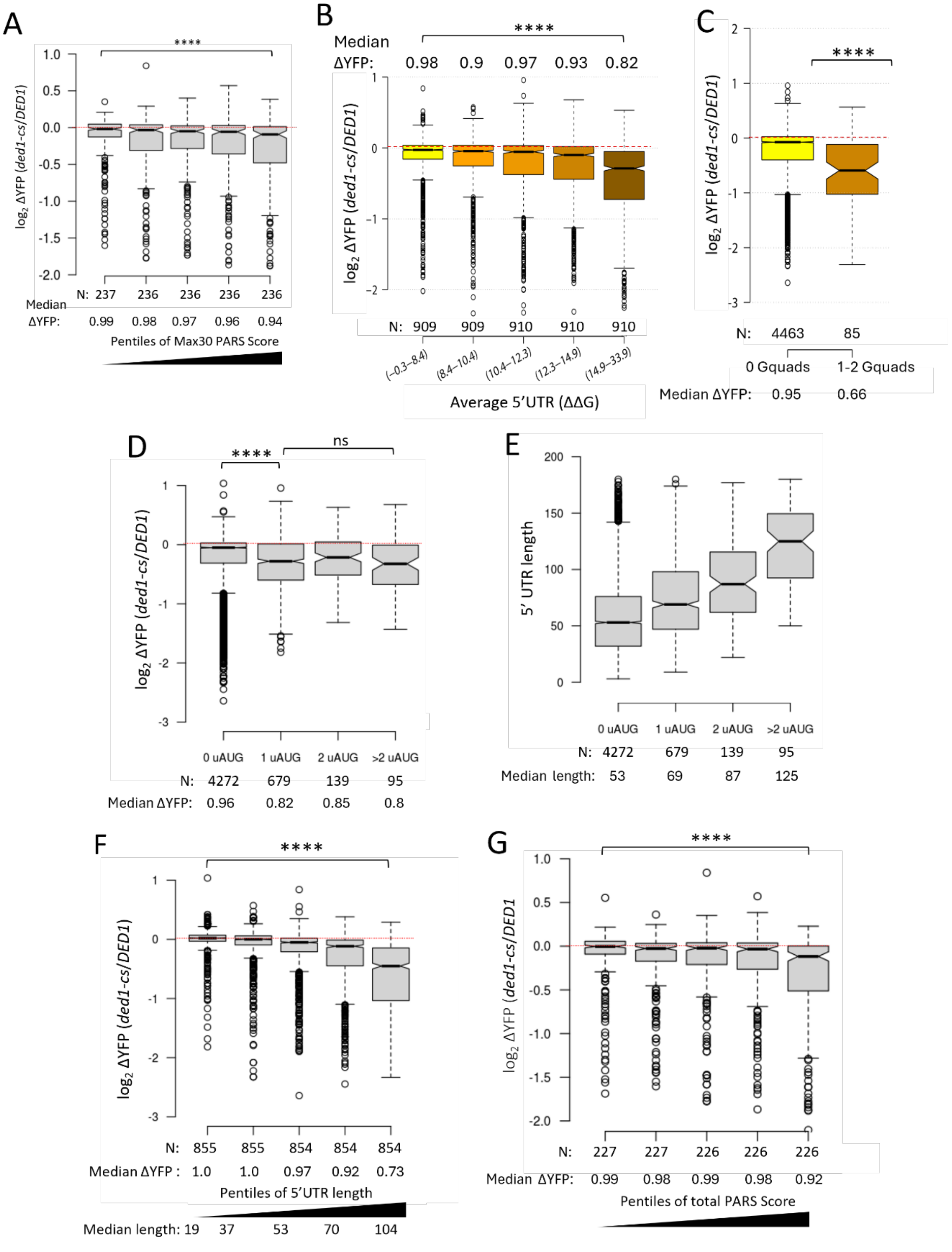

Fig. S12. Evidence that 5'UTR length and secondary structure rather than the presence of upstream AUGs dictate Ded1-dependence of YFP reporter expression in MPRA of most WT

bioRxiv preprint doi: https://doi.org/10.64898/2026.04.10.717766; this version posted April 11, 2026. The copyright holder for this preprint (which was not certified by peer review) is the author/funder. This article is a US Government work. It is not subject to copyright under 17 USC 105 and is also made available for use under a CC0 license.

reporters in the FACS-uORF library. (A) Similar analysis as in Fig. 7D but binned according to the 5'UTR Max30 PARS scores for the subset of 1258 WT YFP reporters with available PARS data. **(B-C)** Notched boxplot of the $\log_2$ changes in reporter expression between *ded1-cs* and *DED1* cells for the subsets of WT YFP reporters binned according to the estimated ΔΔG required to unfold the entire 5'UTR computed with RNAfold (A) or by the number of predicted g-quadruplexes in the 5'UTRs (B) for 4548 of the WT YFP reporters. **(D)** Notched boxplot of the $\log_2$ changes in reporter expression between *ded1-cs* and *DED1* cells for the subsets of WT YFP reporters binned according to the number of AUG codons in the 5'UTRs for which Mut_uORF reporters are represented in the FACS-uORF library. **(E)** Notched boxplot of 5'UTR length for the same bins of WT YFP reporters in (C). **(F-G)** Notched boxplots of the $\log_2$ changes in reporter expression between *ded1-cs* and *DED1* cells for the subset of 4272 WT YFP reporters lacking an AUG codon in the 5'UTR represented by a Mut_uORF reporter in the FACS-uORF library and binned according to reporter 5'UTR length (E), total 5'UTR PARS score (F), or Max30 PARS score. Results of Mann-Whitney U-tests for all panels are summarized as: ****, $P<0.0001$; n.s., not significant, $P>0.05$.

bioRxiv preprint doi: https://doi.org/10.64898/2026.04.10.717766; this version posted April 11, 2026. The copyright holder for this preprint (which was not certified by peer review) is the author/funder. This article is a US Government work. It is not subject to copyright under 17 USC 105 and is also made available for use under a CC0 license.

Supplementary Table S1. YFP reporter plasmid descriptions

	Set I			
Reporter plasmid	Reporter #_uORF disposition	5' UTR Name (Gene, Chromosome, 5'UTR coordinates)	Plasmid name; markers	Description
pRK01	1_WT	YML023C|chrXIII;228708;228664|WT	*pGMENO2YFP mCherry; AmpR, URA3,CEN6*	WT 5'UTR of gene YML023C from the coordinates 228664 to 228708 cloned between the AvrII/BglII sites upstream of the *YFP* CDS.
pRK02	2_WT	YDR072C|chrIV;591470;591344|WT	*pGMENO2YFP mCherry; AmpR, URA3,CEN6*	WT 5'UTR of gene YDR072C from the coordinates 591344 to 591470 cloned between the AvrII/BglII sites upstream of the *YFP* CDS.
pRK03	3_WT	YDR387C|chrIV;1249915;1249821|WT	*pGMENO2YFP mCherry; AmpR, URA3,CEN6*	WT 5'UTR of gene YDR387C from the coordinates 1249821 to 1249915 cloned between the AvrII/BglII sites upstream of the *YFP* CDS.
pRK04	4_WT	YDR186C|chrIV;835546;835492|WT	*pGMENO2YFP mCherry; AmpR, URA3,CEN6*	WT 5'UTR of gene YDR186C from the coordinates 835492 to 835546 cloned between the AvrII/BglII sites upstream of the *YFP* CDS.
pRK05	5_WT	YJR054W|chrX;535988;536055|WT	*pGMENO2YFP mCherry; AmpR, URA3,CEN6*	WT 5'UTR of gene YJR054W from the coordinates 535988 to 536055 cloned between the AvrII/BglII sites upstream of the *YFP* CDS.
pRK06	6_WT	YGL194CA|chrVII;140013;139961|WT	*pGMENO2YFP mCherry; AmpR, URA3,CEN6*	WT 5'UTR of gene YGL194C-A from the coordinates 139961 to 140013 cloned between the AvrII/BglII sites upstream of the *YFP* CDS.
pRK07	7_WT	YIL085C|chrIX;202090;202043|WT	*pGMENO2YFP mCherry; AmpR, URA3,CEN6*	WT 5'UTR of gene YIL085C from the coordinates 202043 to 202090 cloned between the AvrII/BglII sites upstream of the *YFP* CDS.
pRK08	8_WT	YKR089C|chrXI;608417;608365|WT	*pGMENO2YFP mCherry; AmpR, URA3,CEN6*	WT 5'UTR of gene YKR089C from the coordinates 608365 to 608417

bioRxiv preprint doi: https://doi.org/10.64898/2026.04.10.717766; this version posted April 11, 2026. The copyright holder for this preprint (which was not certified by peer review) is the author/funder. This article is a US Government work. It is not subject to copyright under 17 USC 105 and is also made available for use under a CC0 license.

				cloned between the AvrII/BglII sites upstream of the *YFP* CDS.
pRK09	9_WT	YGR010W|chrVII;511469;511544|WT	*pGMENO2YFP mCherry; AmpR, URA3,CEN6*	WT 5'UTR of gene YGR010W from the coordinates 511469 to 511544 cloned between the AvrII/BglII sites upstream of the *YFP* CDS.
pRK10	10_WT	YKR088C|chrXI;605447;605418|WT	*pGMENO2YFP mCherry; AmpR, URA3,CEN6*	WT 5'UTR of gene YKR088C from the coordinates 605418 to 605447 cloned between the AvrII/BglII sites upstream of the *YFP* CDS.
pRK11	11_WT	YMR315W-A|chrXIII;904222;904285|WT	*pGMENO2YFP mCherry; AmpR, URA3,CEN6*	WT 5'UTR of gene YMR315W-A from the coordinates 904222 to 904285 cloned between the AvrII/BglII sites upstream of the *YFP* CDS.
pRK12	12_WT	YDL231C|chrIV;42355;42244|WT	*pGMENO2YFP mCherry; AmpR, URA3,CEN6*	WT 5'UTR of gene YDL231C from the coordinates 42244 to 42355 cloned between the AvrII/BglII sites upstream of the *YFP* CDS.
pRK13	13_WT	YNL042W|chrXIV;548010;548099|WT	*pGMENO2YFP mCherry; AmpR, URA3,CEN6*	WT 5'UTR of gene YNL042W from the coordinates 548010 to 548099 cloned between the AvrII/BglII sites upstream of the *YFP* CDS.
pRK14	14_WT	YEL075W-A|chrV;4732;4869|WT	*pGMENO2YFP mCherry; AmpR, URA3,CEN6*	WT 5'UTR of gene YEL075W-A from the coordinates 4732 to 4869 cloned between the AvrII/BglII sites upstream of the *YFP* CDS.
pRK15	1_Mut	YML023C|chrXIII;228708;228664|228690,228666,AAG	*pGMENO2YFP mCherry; AmpR, URA3,CEN6*	5'UTR of gene YML023C from the coordinates 228664 to 228708 cloned between the AvrII/BglII sites upstream of the *YFP* CDS with AAG uORF start codon mutation at 228690 position.
pRK16	2_Mut	YDR072C|chrIV;591470;591344|591401,591350,AAG	*pGMENO2YFP mCherry; AmpR, URA3,CEN6*	5'UTR of gene YDR072C from the coordinates 591344 to 591470 cloned between the AvrII/BglII sites upstream of the *YFP* CDS with AAG

bioRxiv preprint doi: https://doi.org/10.64898/2026.04.10.717766; this version posted April 11, 2026. The copyright holder for this preprint (which was not certified by peer review) is the author/funder. This article is a US Government work. It is not subject to copyright under 17 USC 105 and is also made available for use under a CC0 license.

				uORF start codon mutation at 591401 position.
pRK17	3_Mut	YDR387C|chrIV;1249915;1249821|1249900,1249828,AAG	*pGMENO2YFP mCherry; AmpR, URA3,CEN6*	5'UTR of gene YDR387C from the coordinates 1249821 to 1249915 cloned between the AvrII/BglII sites upstream of the *YFP* CDS with AAG uORF start codon mutation at 1249900 position.
pRK18	4_Mut	YDR186C|chrIV;835546;835492|835511,835492,AAG	*pGMENO2YFP mCherry; AmpR, URA3,CEN6*	5'UTR of gene YDR186C from the coordinates 835492 to 835546 cloned between the AvrII/BglII sites upstream of the *YFP* CDS with AAG uORF start codon mutation at 835511 position.
pRK19	5_Mut	YJR054W|chrX;535988;536055|536020,536032,AAG	*pGMENO2YFP mCherry; AmpR, URA3,CEN6*	5'UTR of gene YJR054W from the coordinates 535988 to 536055 cloned between the AvrII/BglII sites upstream of the *YFP* CDS with AAG uORF start codon mutation at 536020 position.
pRK20	6_Mut	YGL194CA|chrVII;140013;139961 |139982,AAG	*pGMENO2YFP mCherry; AmpR, URA3,CEN6*	5'UTR of gene YGL194C-A from the coordinates 139961 to 140013 cloned between the AvrII/BglII sites upstream of the *YFP* CDS with AAG oORF start codon mutation at 139982 position.
pRK21	7_Mut	YIL085C|chrIX;202090;202043|202054,AAG	*pGMENO2YFP mCherry; AmpR, URA3,CEN6*	5'UTR of gene YIL085C from the coordinates 202043 to 202090 cloned between the AvrII/BglII sites upstream of the *YFP* CDS with AAG oORF start codon mutation at 202054 position.
pRK22	8_Mut	YKR089C|chrXI;608417;608365|608413,608407,AAG	*pGMENO2YFP mCherry; AmpR, URA3,CEN6*	5'UTR of gene YKR089C from the coordinates 608365 to 608417 cloned between the AvrII/BglII sites upstream of the *YFP* CDS with AAG

bioRxiv preprint doi: https://doi.org/10.64898/2026.04.10.717766; this version posted April 11, 2026. The copyright holder for this preprint (which was not certified by peer review) is the author/funder. This article is a US Government work. It is not subject to copyright under 17 USC 105 and is also made available for use under a CC0 license.

				uORF start codon mutation at 608413 position.
pRK23	9_Mut	YGR010W|chrVII;511469;511544|511478,511508,AAG	*pGMENO2YFP mCherry; AmpR, URA3,CEN6*	5'UTR of gene YGR010W from the coordinates 511469 to 511544 cloned between the AvrII/BglII sites upstream of the *YFP* CDS with AAG uORF start codon mutation at 511478 position.
pRK24	10_Mut	YKR088C|chrXI;605447;605418|605447,AAG	*pGMENO2YFP mCherry; AmpR, URA3,CEN6*	5'UTR of gene YKR088C from the coordinates 605418 to 605447 cloned between the AvrII/BglII sites upstream of the *YFP* CDS with AAG oORF start codon mutation at 605447 position.
pRK25	11_Mut	YMR315W-A|chrXIII;904222;904285|904242,AAG	*pGMENO2YFP mCherry; AmpR, URA3,CEN6*	5'UTR of gene YMR315W-A from the coordinates 904222 to 904285 cloned between the AvrII/BglII sites upstream of the *YFP* CDS with AAG oORF start codon mutation at 904242 position.
pRK26	12_Mut	YDL231C|chrIV;42355;42244|42280,42253,AAG	*pGMENO2YFP mCherry; AmpR, URA3,CEN6*	5'UTR of gene YDL231C from the coordinates 42244 to 42355 cloned between the AvrII/BglII sites upstream of the *YFP* CDS with AAG oORF start codon mutation at 42280 position.
pRK27	13_Mut	YNL042W|chrXIV;548010;548099|548061,548103,AAG	*pGMENO2YFP mCherry; AmpR, URA3,CEN6*	5'UTR of gene YNL042W from the coordinates 548010 to 548099 cloned between the AvrII/BglII sites upstream of the *YFP* CDS with AAG uORF start codon mutation at 548061 position.
pRK28	14_Mut	YEL075W-A|chrV;4732;4869|4848,AAG	*pGMENO2YFP mCherry; AmpR, URA3,CEN6*	5'UTR of gene YEL075W-A from the coordinates 4732 to 4869 cloned between the AvrII/BglII sites upstream of the *YFP* CDS with AAG

bioRxiv preprint doi: https://doi.org/10.64898/2026.04.10.717766; this version posted April 11, 2026. The copyright holder for this preprint (which was not certified by peer review) is the author/funder. This article is a US Government work. It is not subject to copyright under 17 USC 105 and is also made available for use under a CC0 license.

				oORF start codon mutation at 4848 position.

	Set II			
Reporter No.	Reporter #_uORF disposition	5' UTR Name (Gene, Chromosome, 5'UTR coordinates)	Plasmid name; marker	Description
pRK29	1_WT-WT	YIL109C|chrIX;160252;160165|WT	*pGMENO2YFP mCherry; AmpR, URA3,CEN6*	WT 5'UTR of gene YIL109C from the coordinates 160165 to 160252 cloned between the AvrII/BglII sites upstream of the *YFP* CDS.
pRK30	2_WT-WT	YBL032W|chrII;160093;160183|WT	*pGMENO2YFP mCherry; AmpR, URA3,CEN6*	WT 5'UTR of gene YBL032W from the coordinates 160093 to 160183 cloned between the AvrII/BglII sites upstream of the *YFP* CDS.
pRK31	3_WT-WT	YDR481C|chrIV;1420305;1420250|WT	*pGMENO2YFP mCherry; AmpR, URA3,CEN6*	WT 5'UTR of gene YDR481C from the coordinates 1420250 to 1420305 cloned between the AvrII/BglII sites upstream of the *YFP* CDS.
pRK32	4_WT-WT	YHR135C|chrVIII;374419;374310|WT	*pGMENO2YFP mCherry; AmpR, URA3,CEN6*	WT 5'UTR of gene YHR135C from the coordinates 374310 to 374419 cloned between the AvrII/BglII sites upstream of the *YFP* CDS.
pRK33	5_WT-WT	YDL081C|chrIV;310188;310122|WT	*pGMENO2YFP mCherry; AmpR, URA3,CEN6*	WT 5'UTR of gene YDL081C from the coordinates 310122 to 310188 cloned between the AvrII/BglII sites upstream of the *YFP* CDS.
pRK34	6_WT-WT	YKR089C|chrXI;608417;608365|WT	*pGMENO2YFP mCherry; AmpR, URA3,CEN6*	WT 5'UTR of gene YKR089C from the coordinates 608365 to 608417 cloned between the AvrII/BglII sites upstream of the *YFP* CDS.
pRK35	7_WT-WT	YGR086C|chrVII;650725;650617|WT	*pGMENO2YFP mCherry; AmpR, URA3,CEN6*	WT 5'UTR of gene YGR086C from the coordinates 650617 to 650725 cloned between the AvrII/BglII sites upstream of the *YFP* CDS.
pRK36	1_Mut-WT	YIL109C|chrIX;160252;160165|160207,160192,AAG	*pGMENO2YFP mCherry; AmpR, URA3,CEN6*	5'UTR of gene YIL109C from the coordinates 160165 to 160252

bioRxiv preprint doi: https://doi.org/10.64898/2026.04.10.717766; this version posted April 11, 2026. The copyright holder for this preprint (which was not certified by peer review) is the author/funder. This article is a US Government work. It is not subject to copyright under 17 USC 105 and is also made available for use under a CC0 license.

				cloned between the AvrII/BglII sites upstream of the *YFP* CDS with AAG uORF start codon mutation at 160207 position.
pRK37	2_Mut-WT	YBL032W|chrII;160093;160183|160143,160152,AAG	*pGMENO2YFP mCherry; AmpR, URA3,CEN6*	5'UTR of gene YBL032W from the coordinates 160093 to 160183 cloned between the AvrII/BglII sites upstream of the *YFP* CDS with AAG uORF start codon mutation at 160143 position.
pRK38	3_Mut-WT	YDR481C|chrIV;1420305;1420250|1420281,1420254,AAG	*pGMENO2YFP mCherry; AmpR, URA3,CEN6*	5'UTR of gene YDR481C from the coordinates 1420250 to 1420305 cloned between the AvrII/BglII sites upstream of the *YFP* CDS with AAG uORF start codon mutation at 1420281 position.
pRK39	4_Mut-WT	YHR135C|chrVIII;374419;374310|374384,AAG	*pGMENO2YFP mCherry; AmpR, URA3,CEN6*	5'UTR of gene YHR135C from the coordinates 374310 to 374419 cloned between the AvrII/BglII sites upstream of the *YFP* CDS with AAG oORF start codon mutation at 374384 position.
pRK40	5_Mut-WT	YDL081C|chrIV;310188;310122|310172,310151,AAG	*pGMENO2YFP mCherry; AmpR, URA3,CEN6*	5'UTR of gene YDL081C from the coordinates 310122 to 310188 cloned between the AvrII/BglII sites upstream of the *YFP* CDS with AAG uORF start codon mutation at 310172 position.
pRK41	6_Mut-WT	YKR089C|chrXI;608417;608365|608413,608407,AAG	*pGMENO2YFP mCherry; AmpR, URA3,CEN6*	5'UTR of gene YKR089C from the coordinates 608365 to 608417 cloned between the AvrII/BglII sites upstream of the *YFP* CDS with AAG uORF start codon mutation at 608413 position.
pRK42	7_Mut-WT	YGR086C|chrVII;650725;650617|650667,650595,AAG	*pGMENO2YFP mCherry; AmpR, URA3,CEN6*	5'UTR of gene YGR086C from the coordinates 650617 to 650725 cloned between the AvrII/BglII sites

bioRxiv preprint doi: https://doi.org/10.64898/2026.04.10.717766; this version posted April 11, 2026. The copyright holder for this preprint (which was not certified by peer review) is the author/funder. This article is a US Government work. It is not subject to copyright under 17 USC 105 and is also made available for use under a CC0 license.

				upstream of the *YFP* CDS with AAG oORF start codon mutation at 650667 position.
pRK43	1_WT-Mut	YIL109C|chrIX;160252;160165|	*pGMENO2YFP mCherry; AmpR, URA3,CEN6*	WT 5'UTR of gene YIL109C from the coordinates 160165 to 160252 with CAA repeats substituting the structure prone nucleotides (nt.) between 7 to 30 nt. window downstream to the uORF start codon cloned between the AvrII/BglII sites upstream of the *YFP* CDS.
pRK44	2_WT-Mut	YBL032W|chrII;160093;160183|	*pGMENO2YFP mCherry; AmpR, URA3,CEN6*	WT 5'UTR of gene YBL032W from the coordinates 160093 to 160183 with with CAA repeats substituting the structure prone nucleotides (nt.) between 7 to 30 nt. window downstream to the uORF start codon cloned between the AvrII/BglII sites upstream of the *YFP* CDS.
pRK45	3_WT-Mut	YDR481C|chrIV;1420305;1420250|	*pGMENO2YFP mCherry; AmpR, URA3,CEN6*	WT 5'UTR of gene YDR481C from the coordinates 1420250 to 1420305 with CAA repeats substituting the structure prone nucleotides (nt.) between 7 to 30 nt. window downstream to the uORF start codon cloned between the AvrII/BglII sites upstream of the *YFP* CDS.
pRK46	4_WT-Mut	YHR135C|chrVIII;374419;374310|WT	*pGMENO2YFP mCherry; AmpR, URA3,CEN6*	WT 5'UTR of gene YHR135C from the coordinates 374310 to 374419 with CAA repeats substituting the structure prone nucleotides (nt.) between 7 to 30 nt. window downstream to the oORF start codon cloned between the AvrII/BglII sites upstream of the *YFP* CDS.
pRK47	5_WT-Mut	YDL081C|chrIV;310188;310122|	*pGMENO2YFP mCherry; AmpR, URA3,CEN6*	WT 5'UTR of gene YDL081C from the coordinates 310122 to 310188

bioRxiv preprint doi: https://doi.org/10.64898/2026.04.10.717766; this version posted April 11, 2026. The copyright holder for this preprint (which was not certified by peer review) is the author/funder. This article is a US Government work. It is not subject to copyright under 17 USC 105 and is also made available for use under a CC0 license.

				with CAA repeats substituting the structure prone nucleotides (nt.) between 7 to 30 nt. window downstream to the uORF start codon cloned between the AvrII/BglII sites upstream of the *YFP* CDS.
pRK48	6_WT-Mut	YKR089C|chrXI;608417;608365|	*pGMENO2YFP mCherry; AmpR, URA3,CEN6*	WT 5'UTR of gene YKR089C from the coordinates 608365 to 608417 with CAA repeats substituting the structure prone nucleotides (nt.) between 7 to 30 nt. window downstream to the uORF start codon cloned between the AvrII/BglII sites upstream of the *YFP* CDS.
pRK49	7_WT-Mut	YGR086C|chrVII;650725;650617|	*pGMENO2YFP mCherry; AmpR, URA3,CEN6*	WT 5'UTR of gene YGR086C from the coordinates 650617 to 650725 with CAA repeats substituting the structure prone nucleotides (nt.) between 7 to 30 nt. window downstream to the oORF start codon cloned between the AvrII/BglII sites upstream of the *YFP* CDS.
pRK50	1_Mut-Mut	YIL109C|chrIX;160252;160165|160207,160192,AAG	*pGMENO2YFP mCherry; AmpR, URA3,CEN6*	5'UTR of gene YIL109C from the coordinates 160165 to 160252 with CAA repeats substituting the structure prone nucleotides (nt.) between 7 to 30 nt. window downstream to the uORF start codon cloned between the AvrII/BglII sites upstream of the *YFP* CDS with AAG uORF start codon mutation at 160207 position.
pRK51	2_Mut-Mut	YBL032W|chrII;160093;160183|160143,160152,AAG	*pGMENO2YFP mCherry; AmpR, URA3,CEN6*	5'UTR of gene YBL032W from the coordinates 160093 to 160183 with CAA repeats substituting the structure prone nucleotides (nt.) between 7 to 30 nt. window

bioRxiv preprint doi: https://doi.org/10.64898/2026.04.10.717766; this version posted April 11, 2026. The copyright holder for this preprint (which was not certified by peer review) is the author/funder. This article is a US Government work. It is not subject to copyright under 17 USC 105 and is also made available for use under a CC0 license.

				downstream to the uORF start codon cloned between the AvrII/BglII sites upstream of the *YFP* CDS with AAG uORF start codon mutation at 160143 position.
pRK52	3_Mut-Mut	YDR481C|chrIV;1420305;1420250|1420281,1420254,AAG	*pGMENO2YFP mCherry; AmpR, URA3,CEN6*	5'UTR of gene YDR481C from the coordinates 1420250 to 1420305 with CAA repeats substituting the structure prone nucleotides (nt.) between 7 to 30 nt. window downstream to the uORF start codon cloned between the AvrII/BglII sites upstream of the *YFP* CDS with AAG uORF start codon mutation at 1420281 position.
pRK53	4_Mut-Mut	YHR135C|chrVIII;374419;374310|374384,AAG	*pGMENO2YFP mCherry; AmpR, URA3,CEN6*	5'UTR of gene YHR135C from the coordinates 374310 to 374419 with CAA repeats substituting the structure prone nucleotides (nt.) between 7 to 30 nt. window downstream to the uORF start codon cloned between the AvrII/BglII sites upstream of the *YFP* CDS with AAG oORF start codon mutation at 374384 position.
pRK54	5_Mut-Mut	YDL081C|chrIV;310188;310122|310172,310151,AAG	*pGMENO2YFP mCherry; AmpR, URA3,CEN6*	5'UTR of gene YDL081C from the coordinates 310122 to 310188 with CAA repeats substituting the structure prone nucleotides (nt.) between 7 to 30 nt. window downstream to the uORF start codon cloned between the AvrII/BglII sites upstream of the *YFP* CDS with AAG uORF start codon mutation at 310172 position.
pRK55	6_Mut-Mut	YKR089C|chrXI;608417;608365|608413,608407,AAG	*pGMENO2YFP mCherry; AmpR, URA3,CEN6*	5'UTR of gene YKR089C from the coordinates 608365 to 608417 with

bioRxiv preprint doi: https://doi.org/10.64898/2026.04.10.717766; this version posted April 11, 2026. The copyright holder for this preprint (which was not certified by peer review) is the author/funder. This article is a US Government work. It is not subject to copyright under 17 USC 105 and is also made available for use under a CC0 license.

				CAA repeats substituting the structure prone nucleotides (nt.) between 7 to 30 nt. window downstream to the uORF start codon cloned between the AvrII/BglII sites upstream of the *YFP* CDS with AAG uORF start codon mutation at 608413 position.
pRK56	7_Mut-Mut	YGR086C|chrVII;650725;650617|650667,650595,AAG	*pGMENO2YFP mCherry; AmpR, URA3,CEN6*	5'UTR of gene YGR086C from the coordinates 650617 to 650725 with CAA repeats substituting the structure prone nucleotides (nt.) between 7 to 30 nt. window downstream to the uORF start codon cloned between the AvrII/BglII sites upstream of the *YFP* CDS with AAG oORF start codon mutation at 650667 position.

bioRxiv preprint doi: https://doi.org/10.64898/2026.04.10.717766; this version posted April 11, 2026. The copyright holder for this preprint (which was not certified by peer review) is the author/funder. This article is a US Government work. It is not subject to copyright under 17 USC 105 and is also made available for use under a CC0 license.

SUPPLEMENTARY DATA FILES

File S1. Source Data for Figs. 2A-2D. This file lists the TE changes in mORFs and uORFs for all expressed mRNAs in *ded1-cs* versus *DED1* cells from (-CHX) Ribo-seq experiments, determined by DESeq2 analysis (sheets 2-3). The data and analyses used to generate Figs. 2A-D are provided in sheets 4-6.

File S2. Source Data for Figs. 3A-E and Figs. S8B-G. This file lists mRNAs containing unique AUG- initiated uORFs or NCC uORFs along with the data and analyses used to generate Figs. 3A-E (sheets 2-8) and Figs. S8B-G (sheets 9-14) respectively.

File S3. Source Data for Fig. 4B, Figs. 7A-E, Figs. S10A-D, and Figs. S12D-G. This file contains all primary FACS-uORF data for the *DED1* and *ded1-cs* strains and supporting analysis used to generate Figures 4B, 7A-E, S10A-D, and S12D-G.

File S4. Source Data for Fig. 5. This file contains all YFP reporter data and supporting analysis used to generate Figure 5.

File S5. Source Data for Fig. 6 and Fig. S11B-D. This file contains all YFP reporter data and supporting analysis used to generate Figures 6 and S11B-D.

File S6. Source Data for Figs. S1A-D. This file lists the TE changes in mORFs for all expressed mRNAs in response to the *ded1-cs* or *ded1-ts* mutations under +CHX and -CHX conditions. Expression values for all expressed mRNAs obtained from DESeq2 analysis of the corresponding ribosome profiling datasets are listed. Data for +CHX experiments was taken from Sen et al. (1) (GEO file GSE111255).

File S7. Source Data for Figs. S2A-E and Figs. S3A-B. This file lists number of RPF reads aligned to transcriptome in *ded1* mutants and respective *DED1* strains under -CHX and +CHX conditions (sheet 2), TE changes in uORFs for all expressed mRNAs in *ded1* versus respective

bioRxiv preprint doi: https://doi.org/10.64898/2026.04.10.717766; this version posted April 11, 2026. The copyright holder for this preprint (which was not certified by peer review) is the author/funder. This article is a US Government work. It is not subject to copyright under 17 USC 105 and is also made available for use under a CC0 license.

DED1 cells from (-CHX) Ribo-seq experiments, determined by DESeq2 analysis (sheets 3-4). The data and analyses used to generate Fig.S2E and Figs. S3A-B is given in sheet 5 and sheets 6-7 respectively.

File S8. Source Data for Figs. S4A-D: This file lists the changes in TE and center of ribosome density (CRD) of expressed mRNAs in response to the *ded1-cs* or *ded1-ts* mutations under +CHX or -CHX conditions.

File S9. Source Data for Figs. S7A-C and Fig. S8A. This file contains list of mRNAs containing unique AUG-initiated uORFs or NCC uORFs identified by May et al. (5) , Spealman et al. (4), and Zhou et al. (3) (sheets 2-7) used to generate Fig. S7A-C and the data used to generate Fig. S8A (sheet 8).

File S10. Source Data for Figs. S6A-B. This file contains the data to generate Figs. S6A-B.

File S11. Source Data for Fig. S11A. This file contains the 5'UTR DMS MapSeq data from Guenther et al (2) used to generate Fig. S11A.

File S12. Source Data for Figs. S12A-C. This file contains the FACS-uORF used data to generate Figures S12A-C.

bioRxiv preprint doi: https://doi.org/10.64898/2026.04.10.717766; this version posted April 11, 2026. The copyright holder for this preprint (which was not certified by peer review) is the author/funder. This article is a US Government work. It is not subject to copyright under 17 USC 105 and is also made available for use under a CC0 license.

SUPPLEMENTARY REFERENCES

1. Sen, N.D., Gupta, N., S, K.A., Preiss, T., Lorsch, J.R. and Hinnebusch, A.G. (2019) Functional interplay between DEAD-box RNA helicases Ded1 and Dbp1 in preinitiation complex attachment and scanning on structured mRNAs in vivo. *Nucleic Acids Res*.
2. Guenther, U.P., Weinberg, D.E., Zubradt, M.M., Tedeschi, F.A., Stawicki, B.N., Zagore, L.L., Brar, G.A., Licatalosi, D.D., Bartel, D.P., Weissman, J.S. *et al.* (2018) The helicase Ded1p controls use of near-cognate translation initiation codons in 5' UTRs. *Nature*, **559**, 130–134.
3. Zhou, F., Zhang, H., Kulkarni, S.D., Lorsch, J.R. and Hinnebusch, A.G. (2020) eIF1 discriminates against suboptimal initiation sites to prevent excessive uORF translation genome-wide. *RNA*, **26**, 419–438.
4. Spealman, P., Naik, A.W., May, G.E., Kuersten, S., Freeberg, L., Murphy, R.F. and McManus, J. (2018) Conserved non-AUG uORFs revealed by a novel regression analysis of ribosome profiling data. *Genome Res*, **28**, 214–222.
5. May, G.E., Akirtava, C., Agar-Johnson, M., Micic, J., Woolford, J. and McManus, J. (2023) Unraveling the influences of sequence and position on yeast uORF activity using massively parallel reporter systems and machine learning. *Elife*, **12**.
6. Kertesz, M., Wan, Y., Mazor, E., Rinn, J.L., Nutter, R.C., Chang, H.Y. and Segal, E. (2010) Genome-wide measurement of RNA secondary structure in yeast. *Nature*, **467**, 103–107.
7. Kulkarni, S.D., Zhou, F.J., Sen, N.D., Zhang, H.G., Hinnebusch, A.G. and Lorsch, J.R. (2019) Temperature-dependent regulation of upstream open reading frame translation in. *Bmc Biology*, **17**.
